# *Salmonella* Presence and Risk Mitigation in Pet Foods: A Growing Challenge with Implications for Human Health

**DOI:** 10.1111/1541-4337.70060

**Published:** 2024-11-12

**Authors:** Janak Dhakal, Leslie Pearl M. Cancio, Aiswariya Deliephan, Byron D. Chaves, Stephan Tubene

**Affiliations:** ^1^ Department of Agriculture, Food and Resource Sciences University of Maryland Eastern Shore Princess Anne Maryland USA; ^2^ Provincial Science and Technology Office, Davao del Sur Department of Science and Technology XI (DOST XI) Digos City Philippines; ^3^ Kraft Heinz R&D Center Glenview Illinois USA; ^4^ Department of Food Science and Technology University of Nebraska–Lincoln Lincoln Nebraska USA

**Keywords:** Multidrug‐resistant *Salmonella*, outbreaks, prevalence, regulatory measure, transmission

## Abstract

Pet food is increasingly recognized as a significant vehicle for the transmission of foodborne pathogens to humans. The intimate association between pets and their owners, coupled with the rising trend of feeding pets raw and unprocessed foods, contributes substantially to this issue. *Salmonella* contamination in pet food can originate from raw materials and feed ingredients, the processing environment, and postprocessing handling and applications. The absence of standardized postprocessing pathogen mitigation steps in the production of dry kibble and treats, along with the lack of validated heat and chemical interventions in raw pet foods, renders pet food susceptible to contamination by pathogens such as *Salmonella*, *Listeria*, *E. coli*, etc. Pets can then serve as carriers of *Salmonella*, facilitating its transmission to pet owners. Since 1999, there have been over 117 recalls of pet foods due to *Salmonella* contamination in the United States, with 11 of these recalls linked to human outbreaks. Notably, 5 of the 11 human outbreaks involved multidrug‐resistant *Salmonella* strains. Various antimicrobial interventions, including high‐pressure processing, ozone, irradiation, chemical treatments such as organic acids and acidulants, plant‐derived antimicrobials, and biological interventions such as bacteriophages, have proven effective against *Salmonella* in pet foods. This review aims to summarize the prevalence of *Salmonella* in different types of pet foods, identify common sources of contamination, outline reported outbreaks, and discuss control measures and the regulatory framework governing pet food safety.

## 1 INTRODUCTION

Approximately two thirds of households in the United States, totaling around 85 million homes, own at least one pet (Acuff et al., 2021). The global pet food market exceeds US$122 billion, with the U.S. pet food market estimated at around US$ 50 billion in 2021 (Wall, 2023). Consumers spent a total of US$100 billion on pet‐related expenditures in 2021 in the United States alone (Thiel, 2023). With increasing trends in the humanization and premiumization of pets, pet owners’ contact with companion animals, including pets, has become an important source of human pathogens within the household (Ehuwa et al., 2021). Humanization of pets refers to the trend of treating pets like humans and family members. *Salmonella*, *Listeria monocytogenes*, and Shiga toxin‐producing *Escherichia coli* (STEC) have all been found in many types of pet foods—dry and wet, which can potentially cross‐contaminate food contact surfaces, utensils, storage areas, and refrigerators, leading to an increased risk for human transmission of these infectious agents (Weese & Rousseau, 2006).

Pet foods are usually labeled ‘not safe for human consumption’ as they do not have the same microbial safety specifications as human foods. However, due to the close bonds between humans and pets, pet foods are often handled with bare hands, and humans tend to co‐mingle with pets, with some children even taking bites of pet foods (Balachandran et al., 2012). The ingredients used in pet foods come from diverse sources, including by‐products from human‐grade food production systems. The lack of postprocessing pathogen mitigation strategies, the tendency for bulk and loose marketing of pet treats (Adley et al., 2011), and a lack of validated pathogen reduction steps during raw pet food production make these products even more vulnerable to postprocess contamination and cross‐contamination (PFI, 2023). Raw pet foods encompass the most significant section of pet foods with reported *Salmonella* contamination (Table 2), thus neither the Food and Drug Administration (FDA) or the Center for Disease Control and Prevention (CDC) recommend feeding raw diets to pets (FDA, 2018f; CDC, 2024b).

Pet foods are typically classified based on their moisture content: dry, semimoist, and wet (canned) pet food with moisture contents of 5%–12%, 22%–35%, and more than 65%, respectively (Hu, 2016). These foods are processed differently. For example, dry pet food is extruded at high temperatures and pressure; semimoist products are thermally treated and generally make use of humectants for preservations; canned pet foods are commercially sterilized and sealed according to U.S. 21 CR part 113; and raw pet foods are sold and served without any pathogen kill step. Therefore, the prevalence and the route of contamination of each type of food also vary.

Human‐adapted *Salmonella* serotypes are pathogenic to dogs and cats. However, acute clinical cases of salmonellosis are rare in these animals (Sanchez et al., 2002). When clinical cases are seen, they are often associated with exposure to high bacterial loads in puppies and kittens, in which enteritis is common (Sanchez et al., 2002). *Salmonella* could get transmitted from a carrier pet to humans via both direct and indirect routes. The median infectious dose of *Salmonella* in dogs and cats is higher than in humans, which is *ca*. 1000 infectious bacteria (Public Health Agency of Canada, 2011; Sanchez et al., 2002). In a multilaboratory survey conducted among dogs and cats between 2012 and 2014 in 36 U.S. states, Reimschuessel et al. (2017) reported that < 1% (3/542) of cats and 2.5% (60/2,422) of dogs being positive for *Salmonella* with 55% of the positive dogs presenting diarrhea. In a separate report by Ellis and Sanchez (2013), the prevalence of *Salmonella* in healthy dogs and cats was reported to range from 1%–36% and 1%–18%, respectively. *Salmonella* prevalence in pet foods in the United States has been estimated at 0 to 44% in dry pet foods (Pace et al., 1977), 7% to 44.4% in raw pet foods (Jones et al., 2019; Strohmeyer et al., 2006), and 12.5% to 41% in pet treats (Li et al., 2012; White et al., 2003). However, the relatively older finding of the high (44%, *n* = 11/25) prevalence of *Salmonella* by Pace et al. is mostly likely due to the faulty batch of one manufacturer, wherein the researchers reported all 11 positive samples were from one specific manufacturer among samples from four different manufacturers tested. A recent study has shown just 0.42% (1/240) of *Salmonella* prevalence in dry pet foods (Nemser et al., 2014). Healthy dogs that are fed *Salmonella*‐contaminated pet food may shed *Salmonella* in their feces and saliva for up to 7 days (British Small Animal Veterinary Association (BSAVA), 2022; Verma et al., 2007).

With the establishment of a so‐called zero‐tolerance policy, *Salmonella* is considered an adulterant in pet foods (FDA, 2013d). As the number of pet owners continues to rise, so does the demand for commercial pet foods. However, limited information is available on the prevalence and risk mitigation strategies of foodborne pathogens, particularly *Salmonella*, in these products. Therefore, this review summarizes and critically analyzes published information on the prevalence, sources of contamination, transmission routes, conventional and novel antimicrobial intervention strategies, and the regulatory framework around *Salmonella* in pet food safety in the United States.

## 2 REVIEW METHODOLOGY

A systematized literature search was conducted on Google Scholar, Scopus, and PubMed. The inclusion criteria for the search were as follows: (a) any scientific research article or official reports about *Salmonella* in pet foods, (b) mitigation strategies against *Salmonella* in pet foods, (c) reported results on *Salmonella* outbreaks, and (d) investigation on pet food safety. The keywords used for the literature search included “*Salmonella*,” “pet food,” “dog food,” “cat food,” “outbreaks,” “recall,” “pet food safety,” “pathogen control,” “human salmonellosis,” “raw pet foods,” “prevalence,” “sources,” “‘FDA,” and “USDA.” Documents in English were retrieved, and articles were assessed for their relevance based on title and abstract, regardless of publication year. With few exceptions, reports were published between 2000 and 2024.

## 3 PREVALENCE OF *SALMONELLA* CONTAMINATION IN PET FOOD

In general, pet foods and pet treats have higher *Salmonella* prevalence than other animal feed. This could be explained by the fact that animal‐derived ingredients constitute around 60% (w/w) of pet foods compared with only 2% (w/w) in finished animal feeds (Brookes, 2001; Hendriks et al., 1999). This is further supported by findings from the U.S. FDA Center for Veterinary Medicine in 1994, where *Salmonella* prevalence in animal‐derived ingredients was higher (82%) than that of plant‐derived ingredients (37%) (McChesney, 1995). Similarly, a study conducted by Li et al. (2012) indicated that 66.1% and 41.3% of animal‐derived ingredients between 2002–2006 and 2007–2008, respectively, were positive for *Salmonella*. Pet foods, as a possible source of *Salmonella*, were recognized as early as 1955, when the pathogen contaminated 26.5% of 98 dehydrated dog meal samples (Galton et al., 1955). From 1955 to 2024, more studies were conducted to evaluate the presence of *Salmonella* in pet foods, with prevalence ranging from 0 to 80% (Table 1). Although there are many factors contributing to higher prevalence rates, including the number of samples and method of detection, the type of pet foods and the processing treatment appear to be the primary influencers. Table 1 summarizes the available scientific literature with research studies on the prevalence of *Salmonella* in different categories of pet foods.

**TABLE 1:** Prevalence of *Salmonella* in different categories of pet foods

Type of Pet food	Positive/sample (%)	*Salmonella* serotype isolated	Method of detection	Study location and Reference
**Dry Dog Foods**				
Dry Dog foods	26.5% (26/98)	Not specified	Not specified	Not mentioned, Galton et al. (1955), as cited in Pace et al. (1977)
Dehydrated dog food	44% (11/25)	Infantis, Thomasville, Havana, Siegburg, Schwarzengrund, Livingstone, Agona, Senftenberg, Minnesota, Lexington, Johannesburg, Newington	Culture, biochemical and serology	USA, Pace et al. (1977)
Dry preparations	0% (0/27)	Not applicable	Culture and Biochemical	Canada, D'Aoust (1978)
Dried pet food	1% (22/2271)	Typhimurium and three other serovars not specified	Culture, biochemical, and serology	Poland, Wojdat et al. (2004)
Dry pet foods	0% (0/24)	Not applicable	Culture and serotyping	USA, Strohmeyer et al. (2006)
Commercial kibble brands	0% (0/5)	Not applicable	Culture, serotyping	USA, Mehlenbacher et al. (2012)
Dry dog and cat foods	0.42% (1/240)	Not specified	Culture, biochemical, and serology	USA, Nemser et al. (2014)
Dry dog foods	0% (0/36)	Not applicable	Culture, biochemical and serology	Poland, Kazimierska et al. (2021)
Dry pet food	64% (42/66)	Not specified (detected Presumptive *Salmonella* colonies)	Culture	Lebanon, Serhan et al. (2022)
Extruded diets	0% (0/24)	Not applicable	Culture, biochemical, PCR, PFGE	Chile, Solis et al. (2022)
Dry whole‐food cat foods	0% (0/6)	Not applicable	Culture	Poland, Zietara‐Wysocka et al. (2023)
Dry pet foods	0% (0/8)	Not applicable	Culture, biochemical, agglutination, PCR, WGS	Portugal, Ribeiro‐Almeida et al. (2024)
Cooked Kibble Diets	0% (0/24)	Not applicable	Culture, Matrix‐assisted laser desorption‐ionization‐time–of‐flight mass spectrometry (MALDI‐TOF), Whole genome sequencing (WGS)	UK, Morgan et al. (2024)
Dry pet foods	0% (0/27)	Not applicable	Culture	UAE, Hadid et al. (2024)
**Raw Pet Foods (RMBD)**				
Raw meat used in greyhound diets	44.64% (50/112)* 66.03% (70/106)**	Typhimurium, Newport, Agona, Muenster, Anatum, Enteritidis, Schwarzengrund, Bardo, Dublin, Mbandaka, Reading, Senftenberg, Thomasville, Worthington	Culture*, biochemical, serology, DNA probes**	USA, Chengappa et al. (1993)
Homemade biologically appropriate raw food (BARF) dog food	80% (8/10)	Braenderup, Hadar, Schwarzengrund	Culture, serology	Canada, Joffe and Schlesinger (2002)
Commercial raw diets from eight manufacturers	20% (5/25)	Monophasic S. Typhimurium	Culture, and biochemical	Canada, Weese et al. (2005)
Raw meat diet for dogs	7% (17/240)	Reading, Muenster, Cerro, Dublin, Montevideo, Newport, Saint Paul	Culture, serotyping	USA, Strohmeyer et al. (2006)
Commercial raw dog food diets	21% (35/166)	Heidelberg, Hadar, Agona, I:ROUGH‐O:z10:enx, Albert, Mbandaka, IV:ROUGH‐O:‐:‐, Infantis, Thompson, Schwarzengrund, Kentucky, I:ROUGH‐O:r:1,2, Typhimurium, I:4,12:‐:‐, Brandenburg, Meleagridis	Culture, serotyping, and phage typing	Canada, Finley et al. (2008)
Raw pet food diets (frozen, dehydrated, freeze‐dried)	7% (4/60)	4, 12:i:‐, Montevideo, Kentucky, Anatum	Culture, serotyping	USA, Mehlenbacher et al. (2012)
Raw dog and cat foods	7.65% (15/196)	Not specified	Culture, biochemical, and serology	USA, Nemser et al. (2014)
Raw meat‐ based diet (RMBD)	2% (2/88)	Not specified	Culture, PCR	Finland, Fredriksson‐Ahomaa et al. (2017)
RMBD	20% (7/35)	Not specified	Culture, serology	Netherland, Van Bree et al. (2018)
Raw pet food	44.4% (4/9)	Reading, Anatum, Montevideo, Newport	Culture, serotyping, and whole genome sequencing	USA, Jones et al. (2019)
RMBD	7% (4/60)	Rissen, Leeuwarden, Typhimurium, Monophasic *S*. Typhimurium 4,5:i:	Culture, MALDI‐TOF	Sweden, Hellgren et al. (2019)
RMBD (frozen)	0% (0/29)	Not applicable	Culture, biochemical, and serology	Italy, Morelli et al. (2019)
RMBD for dogs	3.9% (2/51)	Monophasic *S*. Typhimurium 4,12:i:‐, London	Culture, serology	Switzerland, Nuesch‐Inderbinen et al. (2019)
Biologically appropriate raw food (BARF) diet	71.4% (15/21)	Not specified	Culture, biochemical, serology	Italy, Bottari et al. (2020)
Raw pet food (frozen and freeze dried)	53% (9/17)	Not specified	Enzyme‐linked fluorescent assay	Thailand, Kananub et al. (2020)
RMBD	33.8% (22/65)	Not specified (detected Presumptive *Salmonella* colonies)	Culture	USA, Cancio et al. (2022)
RMBDs	26.2 % (11/42)	Not specified	Culture, biochemical testing, PCR, PFGE	Chile, Solis et al. (2022)
RMBDs	20% (2/10)	Not specified	Culture, biochemical, serology, ELISA	Germany, Vecchiato et al. (2022)
RMBD (dog food)	12% (7/60)	Infantis, Typhimurium, Schwarzengrund (two isolates were unable to be identified)	Culture, serotyping, PCR	Japan, Yukawa et al. (2022)
BARF for dogs	55.6% (69/124)	Not specified	Culture, biochemical	Peru, Espinoza‐Garate, and Morales‐Cauti (2022)
meat by‐products (MBP) harvested at knackeries (fresh and frozen)	14.8% (77/521)	Dublin, Typhimurium, Monophasic *S*. Typhimurium, Montevideo, Braenderup, Anatum, Agama	Culture, biochemical, and serology	Ireland, McDonnell et al. (2022)
Raw‐frozen	7% (1/14)	1,4,[5],12:i:‐ ST34/cgST142761	Culture, biochemical, agglutination, PCR, WGS	Portugal, Ribeiro‐Almeida et al. (2024)
Pre‐prepared raw diet	4.5% (5/110)	Kottbus, Typhimurium, Indiana, Enteriditis *S*. enterica diarizonae (subsp.)	Culture, MALDI‐TOF, WGS	UK, Morgan et al. (2024)
**Pet Treats**				
Dog Chews	7.8% (184/2369)	Havana,Binza, Montevideo, Caracas, Nima, Bareilly, Senftenberg, Orion, Tennessee, Anatum, Ohio, Stanley, Altona, Nienstadten, Hvittingvoss, Liverpool, Aberdeen, Weltevreden, Typhimurium, Cerro, Give, Lexington, Mbandaka, Rissen, Saint Paul, Newport, Virchow and five unnamed/not determined	Culture, serotyping	England and UK, Willis, 2001
Pig ears	51% (48/94)	Infantis, Typhimurium, Derby, Anatum, Worthington, Ohio, Heidelberg, Brandenberg, California, Bovismorbificans, Mbandaka, Agona, Schwarzengrund,Livingstone, Montevideo, Muenster, Panama, Typhimurium var. Copenhagen, Uganda	Culture, biochemical, and Phage typing, PFGE	Canada, Clark et al. (2001)
Pet treats (lamb, turkey, or beef products)	38% (15/39)	Agona, Agoueve, Banana, Bovismorbificans, Brandenberg, Derby, Havana, Infantis, Mbandaka, Meleagridis, Montevideo, Typhimurium, Typhimurium var. Copenhagen	Culture, biochemical, and Phage typing, PFGE	Canada, Clark et al. (2001)
Dog treats (from pig ears and other animal parts)	41% (65/158)	Anatum, Typhimurium, Infantis, Derby, Ohio, Mbandaka, Bredeney, Worthington, Newport, Muenchen, Freetown, London, Senftenberg, Montevideo, Jerusalem, Heidelberg, Brandenburg, Agona, Grampian, Uganda, Meleagridis, Johannesburg, Gaminara, Orion	Culture, serotyping, phage typing, PFGE	USA, White et al. (2003)
Pet Chews	12 % (36/300)	London, Kentucky, Borreze, Aberdeen, Infantis, Havana, Montevideo, Orion 15+, Senftenberg, Typhimurium, Brandenburg, Enteritidis PT9A, Mbandaka, Montevideo, Ohio	Culture, immuno‐magnetic separation (IMS), biochemical, serology, PFGE	New Zealand, Wong et al. (2007)
Pig ear pet treats	24.5% (25/102)* 28.4% (29/102)^†^	4,5,12:i:‐, Derby, Give, Infantis, Kortrijk, Livingstone, Rissen, Typhimurium	Culture*, serology, phage typing, PFGE, PCR^†^	Ireland, Adley et al. (2011)
Pet treats	12.3%‐ (2002‐2006) 4.8%‐ (2007‐2009)	45 serotypes including, Senftenberg, Montevideo, Mbandaka, Tennessee, Typhimurium, I 4, [5], 1 2:i:‐, Schwarzengrund, Anatum, Agona, Johannesburg, Enteritidis, Havana, Cerro, Oranienberg, Arkansas, Bredeney,Cubana, Derby, Alachua, Hadar, Weltevreden, Amager, Muenchen, Kentucky, Lille	Culture, serotyping	USA, Li et al. (2012)
Pet treats chews (for export)	0.93% (1/108)	Not specified	Culture, biochemical, and serology	Brazil, Galvao et al. (2014)
Jerky‐type treats	0% (0/190)	Not applicable	Culture, biochemical, and serology	USA, Nemser et al. (2014)
Animal‐derived dog treats	2.31% (7/303)	4,5,12:i:–, Rissen, Thompson	Culture, serology, PCR	Japan, Yukawa et al. (2019)
Dried natural dog treats	16% (13/84)	Dublin, Derby, Infantis, Anatum, Monophasic Typhimurium	Culture, MALDI‐TOF, WGS	UK, Morgan et al. (2023)
Dog treats	0% (0/7)	Not applicable	Culture, biochemical, agglutination, PCR, WGS	Portugal, Ribeiro‐Almeida et al. (2024)
**Canned and Cooked Pet Foods**				
Canned pet foods	0% (0/29)	Not applicable	Culture and Biochemical	Canada, D'Aoust (1978)
Cooked open pet foods	19.7% (J. M. Watkinson, personal communication)	Typhimurium, Bredeney, Hadar, Virchow, Agona, Enteritidis, Indiana, Saint Paul, other serotypes	Culture, serotyping, and Phage typing	U. K., Barrell (1982)
Canned pet foods	0 % (0/18)	Not applicable	Culture, biochemical, and serology	Poland, Wojdat et al. (2004)
Canned pet foods	0 % (0/24)	Not applicable	Culture, serotyping	USA, Strohmeyer et al. (2006)
Canned pet foods	26% (26/99)	Not mentioned in the paper (detected Presumptive *Salmonella* colonies)	Culture	Lebanon, Serhan et al. (2022)
Wet pet foods	0% (0/22)	Not applicable	Culture, biochemical, agglutination, PCR, WGS	Portugal, Ribeiro‐Almeida et al. (2024)
**Semi‐moist Pet Foods**				
Moist meat preparations	0% (0/4)	Not applicable	Culture and Biochemical	Canada, D'Aoust (1978)
Semi‐moist dog and cat foods	0% (0/240)	Not applicable	Culture, biochemical, and serology	USA, Nemser et al. (2014)
Semi‐moist pet foods	0% (0/4)	Not applicable	Culture, biochemical, agglutination, PCR, WGS	Portugal, Ribeiro‐Almeida et al. (2024)

*culture method; **DNA Probe; †PCR method

### 3.1 Prevalence of *Salmonella* in dry pet foods

Dry pet foods, also referred to as kibble, are often sold in sealed packages or containers and are typically subjected to a high thermal and pressure treatment that eliminates microorganisms of public health concern and/or reduces them to acceptable levels (Lambertini et al., 2016a). However, there is no standardized postprocessing pathogen mitigation step in dry pet food production (Bianchini et al., 2012, 2014; Lambertini et al., 2016a), making postprocessing steps the main entry points for pathogens in extruded dry pet food. Once contaminated, *Salmonella enterica* serovar Typhimurium was known to survive for up to six months in dry pet food kibble stored at room temperature (Adelantado et al., 2008). Another study reported that it can survive for up to 19 months in dry pet food kibble (Lambertini et al., 2016c).

Unlike raw pet foods or treats, *Salmonella* contamination in dry pet foods is not common due to the high‐temperature treatment of the raw ingredients. Several studies in the United States, Canada, South America, and Europe, with sample size varying from 24 to 36, have analyzed dry pet food and were not able to detect *Salmonella* in their samples (D’ Aoust, 1978; Kazimierska et al., 2021; Strohmeyer et al., 2006). Similarly, in a recently published study from the UK, Morgan et al. (2023) could not detect *Salmonella* from the tested commercial extruded or cooked pet food kibbles (0/24). However, in a relatively large sample‐size study conducted in Poland by Wojdat et al. (2004), who examined 2271 dry food samples, 22 of them (0.97%) were positive for *Salmonella*. Similarly, Nemser et al. (2014) examined dry pet food in the United States and identified a relatively lower prevalence of *Salmonella*, 0.41% (1/240). Contrastingly, in other studies, such as Pace et al. (1977), in the United States, a higher prevalence (11/25, 44%) of *Salmonella* in dry pet foods was detected. However, it is worth noting that the pet food samples tested in the study of Pace et al. (1977) were collected following the infection of a 2.5‐month‐old girl with *S*. Serovar Havana back in 1976, and that ultimately was linked to dehydrated dog food. Similarly, in a recent prevalence study conducted in Lebanon by Serhan et al. (2022), 64% (42/66) of the dry pet food tested were presumptively positive for *Salmonella*. The study tested the samples by culture method, where samples were first selectively enriched in TT and RV broths followed by plating on selective agar (XLD agar), and the presence of black colonies was reported as presumptive positive (hereafter called culture method). The purchased commercial pet food samples in this study originated from different countries which may have gone through long transport, storage, and handling. Not performing the confirmatory test for *Salmonella* presumptive samples was a drawback of this study.

### 3.2 Prevalence of *Salmonella* in semimoist pet foods

Semimoist pet foods are convenient to feed and are palatable, so they are a popular product. The raw materials used in semimoist pet foods, such as meat and animal by‐products, as well as inadequate processing, cross‐contamination, and improper handling and storage conditions, are some of the contributing factors for pathogens entry. The high‐moisture content in semimoist pet foods provides an environment conducive to bacterial and fungal growth. If the product's water activity is not controlled or if it is not properly maintained, pathogens can proliferate. However, semimoist pet foods present a lower risk of *Salmonella* contamination as two studies have documented zero prevalence of said pathogen. As part of a study by the Veterinary Laboratory Investigation and Response Network (Vet‐LIRN), USA, Nemser et al. (2014) analyzed pouch‐packaged semimoist foods and reported zero prevalence (0/240) of *Salmonella*. Ribeiro‐Almeida et al. (2024) were also not able to detect *Salmonella* in commercial semimoist pet foods they have investigated (0/4).

### 3.3 Prevalence of *Salmonella* in wet pet foods

Generally, canned pet foods contain high‐moisture content, which classifies them as wet pet foods. In the case of canned pet foods, they are commercially sterilized and sealed according to U.S. 21 CFR art 113 in hermetically sealed containers (FDA, 2020). The application of heat and the aseptic process prevents the survival and growth of microorganisms, including *Salmonella*. Canned pet foods were examined for *Salmonella* by D'Aoust (1978), Wojdat et al. (2004), and Strohmeyer et al. (2006), and were not able to detect any out of 29, 18, and 24 samples tested, respectively.

In a study conducted in Manchester, UK (Barrell, 1982), *Salmonella* prevalence in cooked open pet foods was reported as high as 26% (26/99). Similarly, in a study by Serhan et al. (2022) in Lebanon, 99 canned foods were examined, and they isolated presumptive *Salmonella* colonies from 26 (26%) samples. However, although the former conducted serotyping, the latter did not conduct the confirmatory test of the colonies, leaving a space to assume that the true *Salmonella*‐positive samples may be lower. This is in contrast with a recent study from Portugal, where 22 cooked wet pet food samples were tested for *Salmonella*, and all of them were reported negative (Ribeiro‐Almeida et al., 2024).

### 3.4 Prevalence of *Salmonella* pet treats and chews

Dogs and cats are often provided with treats in addition to their basic foods. These treats and chews are considered complementary products (Kepinska‐Pacelik & Biel, 2021). In particular, dogs have conditional cravings for biting, and chews are provided to prevent them from damaging home furniture and appliances. These treats and chews often contain animal by‐products or animal‐derived products. Some examples of such products are beef jerky, animal ears, trachea, tendons, masseters, fish meal, blood meal, and animal fat (Kepinska‐Pacelik & Biel, 2021). Generally, after manufacturing, pet treats undergo a dehydrating step to reduce and bring the moisture content to a desirable level, making it unlikely for *Salmonella* and other pathogens to grow (Lambertini et al., 2016a) unless the product is abused in terms of high moisture or temperature. Often, such animal‐origin chews and treats are not processed and are frequently sold as open and loose in bulk bins (Adley et al., 2011), making them more prone to pathogenic contamination. Unlike regular pet foods, pet treats are not served in the food bowls nor delivered using scoops or spoons. Treats are held by bare hands (direct human contact), posing another level of threat from pathogen transmission amongst the handlers who are unaware of the possible health risks associated with the contaminated pet treats.

A higher prevalence of *Salmonella* in pet treats has been reported in several studies. Clark et al. (2001) conducted a nationwide survey analyzing 94 pig ears and 39 pet treats in Canada and reported 51% (48/94) and 38% (15/39) *Salmonella* prevalence, respectively. Another study in the United States by White et al. (2003) explored 26 domestic and 132 imported pet treats and reported 41% (65/158) *Salmonella*‐positive samples. It is important to note here that both investigations sprung from the incidence of *S*. Infantis infections in humans in Alberta, which was later associated with pig ears as pet treats. Similarly, Adley et al. (2011) tested 102 pet treats from Limerick City, Ireland, and found 24.5% (25/102) *Salmonella*‐positive samples using the culture method and a higher positive rate of 28.4% (29/102) upon PCR confirmation. Notably, in this study, all the positive samples originated from a single distributor. However, the authors did not clarify whether the samples were from the same batch or from different batches. It is likely that if the positive samples were from one particular batch from one single distributor, it could be a contamination issue. On the other hand, in a study conducted in Brazil by Galvao et al. (2014), only 0.93% (1/108) of the pet treats were *Salmonella* positive. However, the limitation of this study was that these samples were obtained from only one supplier that produced pet treats for export. The low *Salmonella* prevalence in these products could be linked to good manufacturing practices and strict implementation of microbiological quality parameters, among others. Li et al. (2012) conducted surveillance of finished feeds, feed ingredients, supplements for pets, pet foods, and pet treats to monitor the trend of *Salmonella* contamination in animal feeds over different periods. It was observed that the prevalence of *Salmonella* in pet foods and pet treats both declined from 13% and 12.3% in 2002–2006 to 9.8% and 4.8% in 2007–2009, respectively. Although the pet food samples were not defined in more detail, the results showed that there is a higher *Salmonella* prevalence in pet foods than in pet treats. Similarly, in a study by the Vet‐LIRN, *Salmonella* was not detected in pet treats (0/190) (Nemser et al., 2014). In a recent study by Morgan et al. (2023), 16% (13/84) of commercially available dry pet treats in the UK were positive for *Salmonella*. When the treats were traced, they found some of the treats were unpackaged with no label, some were individually wrapped, some were delivered in boxes as loose treats, and some in clear plastic bags without labels.

### 3.5 Prevalence of *Salmonella* in raw pet food/raw meat‐based diets

Pet food is considered raw when it contains meat, bones, organs, and/or eggs, sometimes with vegetables that have not been cooked or treated for safety (PFI, 2024). However, because no forms of cooking are employed in raw pet food, nonthermal interventions such as the freeze‐drying process and high‐pressure processing (HPP) may be considered. Despite the disagreement between pet owners and veterinarians in terms of nutrition and public health (Freeman & Michel, 2001; LeJeune & Hancock, 2001; Turnbull, 2003), there is a rising appeal of raw meat‐based diets (RMBD) among pet owners as anecdotal reports on this type of pet food showcase it as a natural diet with potential health benefits for pets (Nuesch‐Inderbinen et al., 2019). Pet owners may believe that nonprocessed meat‐based diets are healthier and natural choices for their pets (Morgan et al., 2023). It is estimated that about 15% to 25% of dogs and 10% of cats are regularly fed RMBD (Stogdale, 2019). However, another study claimed that approximately 60% of pet owners feed their pets completely or partially RMBD (Ahmed et al., 2021). RMBD are also commonly fed among racing greyhounds and sled dogs (LeJeune & Hancock, 2001). However, the health benefits of the RMBD are not scientifically supported, and a serious food safety concern exists due to the natural microbiological loads of said products.

RMBD can be prepared in various forms, including frozen, fresh, or freeze‐dried options. Commercial raw pet foods are mostly made from a combination of raw meat (beef, chicken, duck, lamb, rabbit, veal, venison, etc.) and offal (hearts, liver, gizzards, etc.), fruits, vegetables, grain, eggs, etc. (Hoelzer et al., 2011; Nuesch‐Inderbinen et al., 2019; Raditic, 2021). All of these products are known to be vehicles for *Salmonella* transmission (Freeman et al., 2013). For example, 45% of commercial raw meat diets used in pet foods fed to greyhound were *S*. Typhimurium–positive (Chengappa et al., 1993).

Due to the public health threat raw pet food poses, numerous studies have been conducted to evaluate the microbiological quality of RMBD. We identified 23 *Salmonella* prevalence studies in raw pet foods dating back to 2002, with the majority of them published between 2012–2023. Seven studies focused on analyzing RMBD specifically designed for dogs, whereas the remaining studies examined RMBD intended for pets in general. The prevalence of *Salmonella* in raw pet foods greatly varied from 0–80%. The U.S. FDA cautions the public from feeding raw pet food diets due to *Salmonella* and other associated pathogens (FDA, 2019b).

Mehlenbacher et al. (2012) performed a study on frozen, dehydrated, and freeze‐dried raw pet foods purchased locally in Minneapolis and St. Paul area in Minnesota, USA. They reported 7% (4/60) positive for *Salmonella* serovars 12:i:‐, Montevideo, Kentucky, and Anatum. It was also found that 52% of samples (31/60) were subjected to treatment such as dehydration, freeze‐drying, or HPP, and *Salmonella* was detected in unprocessed samples. It is worth noting here that all the serovars isolated were multidrug‐resistant (MDR). Similarly, two different studies with larger sample sizes were conducted in the United States, one in Colorado by Strohmeyer et al. (2006) and the other at a multistate level by Nemser et al. (2014). They respectively found a similar *Salmonella* prevalence rate of 7.08% (17/240) and 7.65% (15/196) in commercial raw pet foods. The former used raw meat diets composed of beef, lamb, chicken, or turkey meat produced by seven manufacturers. In contrast, the latter collected commercial feed samples from different states within the United States and processed them in six different laboratories. In a separate study, Cancio (2022) analyzed selected raw pet foods in the United States. They recorded presumptive *Salmonella* colonies in 33.8% (22/65) of the RMBD, most of which were blends of skeletal muscles, offal, and edible bones.

In a study conducted in Canada, Finley et al. (2008) reported that approximately 21% of the raw pet food diets are positive for *Salmonella*. Joffe and Schlesinger (2002) investigated homemade raw pet food in Canada and reported 8/10 (80%) samples as *Salmonella*‐positive. The researchers also reported that 3/10 (30%) dogs fed the contaminated raw pet food shed the *Salmonella* serovars in their stool. Another study in Canada, which analyzed 25 raw pet foods (24 frozen, 1 freeze‐dried) originating from eight different manufacturers, identified 20% (5/25) of the samples as *Salmonella*‐positive (Weese et al., 2005). Finley et al. (2008) reported a much higher *Salmonella* prevalence of 21% (35/166) among RMBD sold in Canada. Most of the samples (*n* = 161) had poultry meat as the main ingredient or as one of the two meat ingredients.

Similarly, in a study conducted in the Netherlands, 20% (7/35) of commercial raw pet foods representing eight brands from 14 retailers were positive for *Salmonella* (van Bree et al., 2018). In contrast, a study conducted on commercial raw pet foods composed of domestic beef (43%), poultry (41%), and pork (27%) in Finland observed a relatively lower *Salmonella* prevalence of 2% (2/88) (Fredriksson‐Ahomaa et al., 2017). In an interesting finding by Morelli et al. (2020) in Italy, none of the 29 raw pet food samples tested were positive for *Salmonella*. The raw pet foods were laboratory‐manufactured (raw ingredients were purchased, and raw pet food was formulated in the laboratory) utilizing meat from beef, turkey, chicken, horse, lamb, salmon, horse, and duck. Considering the composition of raw meat, it is unusual that the researchers could not detect a single positive sample despite the culture, biochemical tests, and serology methods for *Salmonella* detection. Studies in Sweden, Switzerland, and Italy also showed a lower *Salmonella* prevalence of 7%, 3.9%, and 7.14%, respectively, when utilizing culture, biochemical, and/or serological testing methods (Bottari et al., 2020; Hellgren et al., 2019; Nuesch‐Inderbinen et al., 2019).

Most of the studies on raw pet food were conducted in the United States, Canada, and Europe, possibly due to the dense pet population and premium pet care trends. Studies on the prevalence of *Salmonella* in RMBDs in Asia and South America are also available. In Thailand, commercial raw pet foods belonging to 12 brands (15 frozen and 2 freeze‐dried) were tested for *Salmonella* prevalence and found that 53% (9/17) of the frozen and freeze‐dried raw pet food was positive for *Salmonella* using enzyme‐linked fluorescent assay technology (Kananub et al., 2020). Similarly, Yukawa et al. (2022) in Japan investigated 60 commercial raw pet food samples from six different brands from the Okayama and Osaka regions and reported the presence of *Salmonella* in 12% (7/60) of the samples. The serovars isolated were Infantis, Typhimurium, and Schwarzengrund, and many of them were MDR *S*. Infantis, which is an emerging concern in the poultry industry in the United States and Europe. In Chile, Solis et al. (2022) tested 31 commercial and 11 homemade raw pet foods (RMBD) and reported *Salmonella* in 11/42 (26.2%) of the samples. In the study, chicken meat was the main ingredient in 6 of the 11 samples that were positive for *Salmonella*. Finally, a recent survey by Morgan et al. (2024) reported that 4.5% (5/110) of pre‐prepared raw pet food diets were *Salmonella* positive.

## 4 SOURCES OF *SALMONELLA* CONTAMINATION IN PET FOODS

### 4.1 Ingredients and raw materials

**FIGURE 1:**
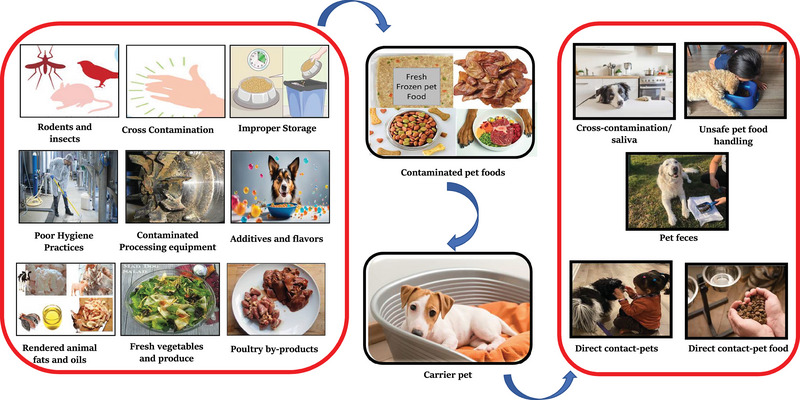
Common sources of pet food contamination and ways humans acquire *Salmonella* from to pet foods.

Because *Salmonella* spp. can be found in dust, soil, rodents, livestock, animal housings, and farm and agriculture products such as grains and meat ingredients, it can easily gain access to and contaminate the pet food production and supply chain at multiple points (Figure 1). Contaminated food ingredients are one of the major sources of *Salmonella* in final pet food products, especially in RMBD. The most common ingredient categories in pet foods include poultry, meat‐ and plant‐based products, additives, enzymes, rendered fat and oils, vitamins, and others (AAFCO, 2024a). The RMBD usually contains skeletal muscle, fat, cartilage, internal organs, and bones of farm animals (poultry, pork, and ruminants), horses, game, and fish (Fredricksson‐Ahomaa et al., 2017). There is an increased risk for contamination in RMBD, given that the raw materials do not generally involve a heat processing or kill step.

Rendered meat and rendered animal products are commonly used in pet foods. Some animal products undergo rendering, a process where raw animal tissues are subjected to heat application, moisture extraction, and fat separation (Meeker & Hamilton, 2006). The animal protein by‐products could be meat and bone meal, meat meal, blood meal, poultry by‐product meal, poultry meal, feather meal, fish meal, etc. In a study by Kinley et al. (2010), *Salmonella* was detected in 8.7% of the 150 meal samples from various rendering companies across the United States. A year‐long study by Jiang (2016) also observed *Salmonella* in 8.3% (731/8,783) of the analyzed samples of rendered products across the United States and Canada. Animal offal and a variety of meats are other major components of pet food products. Offal is also known as a variety of meat which excludes muscle of bones, and mostly comprises internal organs, such as the heart, liver, kidney, tongue, gizzards, etc. In a study in the United States, 59.4% (148/249) of chicken liver, a major component in raw pet food, was found *Salmonella* positive in samples from retail stores in Delaware, New Jersey, and Pennsylvania (Jung et al., 2019). In Egypt, 13.88%, 11.11%, and 6.25% of chicken gizzard, liver, and breast, respectively, tested positive for *Salmonella* (Abd El‐Aziz, 2013). A higher prevalence of *Salmonella* in chicken giblets in samples from Thailand and Ethiopia was recorded at 86% (190/221) and 42% (24/57), respectively (Boniphace, 2001; Jerngklinchan et al., 1994).

### 4.2 Processing environment

The pet food processing environment itself can harbor and become a continuous source of *Salmonella*. Cross‐contamination can occur if surfaces, equipment, or utensils come into contact with *Salmonella*‐contaminated materials or when employees handle food without proper sanitary protocol. If not cleaned and sanitized adequately and regularly, processing equipment (grinders, mixers, conveyors, etc.), floors, and surfaces can become a niche for *Salmonella*, for example, biofilm formation and maturation leading to continuous shedding of *Salmonella*. Cracks, crevices, and hard‐to‐reach areas of equipment provide a conducive environment for *Salmonella* to form a biofilm, which, once mature and ruptured, becomes a regular source of contamination in the supply chain. *Salmonella* exposed to improper or sublethal sanitizing agents are known to be a higher biofilm former (Dhakal et al., 2019a).

There have been reports that *Salmonella* outbreaks in pet foods were linked to contaminated processing environments. One prominent case happened in 2007 when contaminated dry pet foods led to 79 cases of *S*. Schwarzengrund infection in humans. During the investigation, the Pennsylvania Department of Health eventually traced the source to the processing environment in the enrobing and flavoring room of the manufacturing plant (Behravesh et al., 2010). Similarly, another notable pet food‐related outbreak was that of *S*. Infantis in 2012, leading to 49 cases in 20 U.S. states and Canada. During the investigation, dry dog foods were produced in a single pet food production facility in South Carolina, which was linked to the outbreak (CDC, 2012b). Further, the outbreak strain of *S*. Infantis was also isolated from a pet owner's opened pet food bag and unopened dry dog food from retail stores. The U.S. FDA investigation found a plant‐wide contamination in the manufacturing facility, leading to one of the largest pet food recalls in recent history. The inspectors pointed out various shortcomings in the facility, such as failure in the provision of washing and sanitizing facilities within the plant, breakdown in Sanitation Standard Operating Procedures (SSOP) and preventive maintenance program implementation, and inadequate steps to ensure that processing procedures would not contribute to contamination.

Potential sources of contamination in a processing facility include birds (feces and feathers) entering via air vents, traffic patterns, pests, rodents, etc. (Carrion & Thompson, 2013). In facilities where pet food is stored, pests like insects and rodents may access improperly sealed containers or packaging, leading to contamination of the contents with *Salmonella* (Leiva et al., 2019). They can also get access to the food during transportation if it is not properly sealed and protected. Finally, temperature and relative humidity in the processing environment are critical factors to restrict microbial growth in the environment and raw materials. High temperature and humidity may favor bacterial and mold growth. Similarly, moist floors and environments support bacterial growth and proliferation, putting the food production chain at a higher contamination risk.

### 4.3 Postprocess contamination

Considering the high temperature and pressure extrusion as a potent physical antimicrobial intervention, the *Salmonella* presence in dry pet foods is almost always attributed to postprocessing contamination. The incorporation of flavor and coating agents is suspected to be the major step for pathogen recontamination (Dhakal et al., 2019b). Although it was not specified, in the *S*. Schwarzengrund outbreak (2008–2012) in humans mentioned above, the materials sprayed in the finished products were suspected to be contaminated with *Salmonella* (KuKanich, 2011). The lack of standardized food safety protocols and testing methods on the final products could contribute to a delay in identifying the source of contamination and conducting root‐cause analysis to prevent recurrence.

Additionally, packaging and handling could become a potential source of *Salmonella* contamination for semimoist and dry pet foods. Because there is no further kill step after the coating and drying in dry pet food, downstream operations such as oil and fat coating and flavor addition could be a major point of contamination in dry extruded pet foods. One of the notable cases of postprocessing contamination or faulty handling leading to *Salmonella* contamination in raw turkey pet products was linked to the 2017–2019 outbreak of *S*. Reading. Upon investigation, the Iowa Department of Health concluded that the turkey products were improperly prepared and handled, ignoring the USDA FSIS guidelines, and were not held at the appropriate temperature to prevent pathogen growth (Hassan et al., 2019). Compliance with Good Manufacturing Practices (GMP), appropriate process controls and safety plans, proper personal hygiene, and the use of proper packaging material are other vital considerations to ensure the quality and safety of pet foods. GMPs are fundamental to pet food safety, and their proper implementation is the basis of risk management. Any *Salmonella* prevalence in the finished product is indicative of a deviation in their food safety system (Leiva et al., 2019).

Cross‐contamination can also occur during storing and handling of pet food in retail spaces. Pet treats, for example pig ears, are often sold loose in bulk bins in pet shops. Pig ears sold in bulk bins in Ireland were found positive for *Salmonella* (Adley et al., 2011). A study by Finley et al. (2006) in Canada reported that natural pet treats were sold in bulk bins without any packaging material or instructions available to buyers, posing a threat of external contamination via birds, pests, and other vectors. Therefore, to minimize external contamination, pet treats are recommended to be individually packaged and irradiated (KuKanich, 2011)

## 5 TRANSMISSION FROM PETS TO HUMANS

Typically, young animals exhibit higher susceptibility to enteric‐type infections, with the potential for the infection to progress to a systemic level in more severe cases. In contrast, adult animals are more prone to having asymptomatic infections (Carrion & Thompson, 2013). Pets with healthy immune systems or those infected with low infectious doses of the organisms, such as that from contaminated pet foods, usually remain asymptomatic or experience only mild, temporary illnesses. *Salmonella*, a known zoonotic organism can be transmitted from animals to humans and vice‐versa. Transmission from pets to humans is mostly through the handling of contaminated pet foods or by contact with carrier pets. Occasionally, there are cases where *Salmonella*‐contaminated feed leads to severe illnesses in pets. For example, two septicemic cats presented at the Tifton Veterinary Diagnostic and Investigational Laboratory (TVDIL) at the University of Georgia's College of Veterinary Medicine were found to be fed a raw beef diet contaminated with *S*. Newport (Stiver et al., 2003). At the lower level of *Salmonella*, pets act as carriers but could become shedder at home.

### 5.1 Household interactions with pets

The increasing trend of humanization of pets and the close relationship between pets and their owner exposes humans to pathogens, such as *Salmonella*. Companion animals spend much of their lives indoors and in intimate contact with their owners. Household interactions, such as direct and/or indirect contact with pets, direct and/or indirect contact with contaminated pet food, unsafe pet food handling, and/or pet feces are some of the potential routes that pet owners can acquire *Salmonella* from pets (Lambertini et al., 2016b). Figure 1 shows some of the common ways humans can acquire *Salmonella* from their pets.

After consumption, pet food comes in contact with the pet's jowls, whiskers, nose, mouth, and tongue. Pet owners generally do not clean pets’ mouths and faces after they eat. When pets come in direct contact with the owners, including children and the elderly, play, sleep with, hug, and lick, they could potentially transmit foodborne pathogens to the owners. Additionally, it is not unusual that kids and toddlers at home often nibble and eat pet foods from their bowls while playing with pets. A four‐month‐old infant in Japan with symptoms of diarrhea was diagnosed with *S*. Virchow in his stool. Upon investigation, the infectious *Salmonella* serovar was traced back to their household dog, which was a carrier for the pathogen (Sato et al., 2000). Another example of contact with contaminated pet food leading to human infection was reported by Hassan et al. (2019), where two household children were infected with *S*. Reading, which was sourced back to contaminated raw turkey pet food. Pets that are fed raw pet food diets tend to shed increased pathogen levels in their feces (Weese et al., 2005), and RMBD is considered a risk factor for fecal carriage of *Salmonella* by pets (Kaindama et al., 2021).

Indirect contact, such as interactions within a common environment between humans and pets, within the environment where the pet lives, or items used by the pet, such as their toys, food bowls, and grooming tools, could also lead to human salmonellosis. Additionally, this could also be due to unsafe pet food handling, such as improper storage, not cleaning the pet food bowls before and after feeding, and inadequate handwashing and sanitizing during meal preparation. Weese and Rousseau (2006) tested *Salmonella* recovery and survival from common household plastic and stainless‐steel feeding bowls after adding 2 g of meat inoculated with a 5‐log *S*. Copenhagen. The inoculated foods were wiped with a gloved hand, leaving a thin layer of residue, and bowls were allowed to dry at room temperature for 1 h. Following this, the bowls were cleaned using warm water, rinse, soap, bleach, dishwater, etc. The findings were a little unusual that none of the methods, even scrubbing followed by bleach soap, was effective in removing total *Salmonella* from the bowls, with 33% of stainless‐steel bowls and 50% of plastic bowls positive for *Salmonella*. Meanwhile, with both warm water rinse and rinse followed by a scrub, 100% of the stainless‐steel bowl and 92% of the plastic bowl were still positive for *Salmonella*. Similarly, pet feces could also be a potential source of contamination, especially when handling animal waste and if fecal shedding frequently occurs inside the household.

When pets consume *Salmonella*‐contaminated foods, they tend to become carriers. Finley et al. (2008) studied the risk associated with feeding *Salmonella*‐contaminated commercial raw food diets and found out that seven of the 16 exposed dogs shed *Salmonella* after 1 to 7 days of consumption. The dogs fed *Salmonella*‐free diets did not shed *Salmonella* in feces. Additionally, chances of *Salmonella* in fecal shedding are higher in dogs fed with a raw meat diet as compared with conventional diets (Runesvard et al., 2020; Viegas et al., 2020). Therefore, household interaction with carrier pets poses a threat of salmonellosis to humans.

### 5.2 Population at risk

The 2023 human salmonellosis outbreak from a Texas manufacturer's dry pet food was the prime example of how the young and infants are the most vulnerable population, where 86% of the reported ill population were children one year of age or younger (CDC, 2024a). Humans can easily acquire pathogens either from handling contaminated pet foods or from pets. For immunocompromised individuals, salmonellosis was also noted as one of the common zoonotic diseases that can be acquired from pets (Hemsworth & Pizer, 2006). Although the Centers for Disease Control and Prevention recommend washing hands for 20 seconds after handling pet foods and keeping away children below five years from pet foods, treats, and supplements, the extent of adherence of pet owners cannot be ascertained. Feeding RMBDs to pets is also not recommended by the FDA and CDC (FDA, 2018f; CDC, 2024b). These agencies warn of the danger of such practice but also provide some recommended measures to follow if the owners opt to feed RMBDs.

## 6 PET FOOD RECALLS AND *SALMONELLA* OUTBREAKS LINKED TO PET FOODS

Once introduced, *Salmonella* can persist within the pet food matrix due to its ability to survive in low‐moisture environments and resist typical processing conditions (Finn et al., 2013). Any potential cases of *Salmonella* contamination in pet foods that may or may not lead to an outbreak or human cases are investigated by the U.S. FDA in collaboration with the U.S. CDC and State Departments of Agriculture. The confirmed or suspected *Salmonella* contamination is usually followed by voluntary recalls by the manufacturers. Between 2003 and 2022, 859 *Salmonella*‐linked recalls were associated with pet food and constituted 24% of the total pet food recalls, and 85% of total bacterial pathogen‐linked pet food recalls (Debeer et al., 2023). It should be noted here that these recall data include food ingredient recalls, not just pet foods. Timely recalls help minimize the possibility of human illnesses. Table 2 provides a comprehensive and up‐to‐date list of pet food recalls that are contaminated or suspected to be contaminated with *Salmonella*. Additionally, the table identifies any pet food linked to *Salmonella* outbreaks in human outbreaks.

**TABLE 2:** *Salmonella*‐linked Recalls and Outbreaks Linked to Pet Foods

Date^†^	Brand‐Names	Product‐Description	Pathogen(s) identified	Human Illness	Company‐Name	References	Pet Food Type
1999	Retail stores in US and Canda	Pig ear dog Treat	*Salmonella* Infantis*	30 illnesses	Multiple brands	Clark et al. (2001); White et al. (2003); FDA (2024d)	Treat
2002	Not specified	Pet Treats	*S*. Newport PT 14* (MDR)	5 cases. “Patient Zero” was a 1‐month‐old infant.	Texas‐based company	Pitout et al. (2003); Finley et al. (2006)	Treat
2005	USA & Canada‐British Columbia (BC) manufacturing plant	Pet Treats for cats and dogs	*Salmonella* Thompson, Cerro, and Meleagridis*	9 culture confirmed cases in Washington and west Canada	Washington and British Columbia (BC) manufacturing plant	CDC (2006); Finley et al. (2006)	Treat
16‐02‐2007	Wild Kitty Cat Food All Natural, Frozen Cat Food	All Natural, Frozen Cat Food‐ Raw Chicken, Raw Duck and Raw Tuna	*Salmonella* spp.		Wild Kitty Cat Food, Inc	FDA (2007c)	RMBD
09‐03‐2007	Berkley & Jensen	Pig ear dog Treat	*Salmonella* spp.		BJ's Wholesale Club	FDA (2007a)	Treat
16‐04‐2007	A.B. Dog Chew	Dog chew	*Salmonella* spp.		T.W. Enterprises	FDA (2007b)	Treat
08‐08‐2008	PEDIGREE® Complete Nutrition	Small Crunchy Bites	*Salmonella* spp.		Mars Petcare	FDA (2008a)	Dry
12‐09‐2008	Ol' Roy Dog Food, Pedigree Dog Food, Special Kitty Cat Food. etc.	Dry Pet Food Product	*Salmonella* Schwarzengrund*	79 human cases in 21 states	Mars Petcare	FDA (2008b); Deasy et al. (2008); Behravesh et al. (2010); Kukanich (2011)	Dry
10‐03‐2009	Alaska Canine Cookies	Peanut Butter, Power Bone and Carrot Cake flavors of Canine Cookies	*Salmonella* spp.		Alaska Canine Cookies	FDA (2009)	Treat
12‐04‐2010	Cetyl M	Joint Action Formula for Dogs	*Salmonella* spp.		Response Products	FDA (2010h)	Fatty acid supplement
11‐02‐2010	Nature's Variety	Raw Frozen Chicken Diets for dogs and cats	*Salmonella* spp.		Nature's Variety	FDA (2010f)	RMBD
18‐06‐2010	Natural Balance	Sweet Potato and Chicken Dry dog food	*Salmonella* spp.		Natural Balance Pet Food, Inc.	FDA (2010e)	Dry
22‐06‐2010	Pro‐Pet	Adult Vitamin Supplement tablets for dogs	*Salmonella* spp.		United Pet Group	FDA (2010k)	Vitamins
01‐07‐2010	Feline's pride	Raw food with ground bone for cats and kittens, Natural Chicken Formula	*Salmonella* spp.		Feline Pride	FDA (2010b)	RMBD
02‐07‐2010	Pro‐pet, Excel and other Private Label brands	Adult Vitamin Supplement tablets for dogs	*Salmonella* spp.		United Pet Group	FDA (2010j)	Vitamins
02‐07‐2010	Merrick	Beef Filet Squares for Dogs	*Salmonella* spp.		Merrick Pet Care, Inc.	FDA (2010d)	Treat
15‐07‐2010	Feline's pride	Raw food with ground bone for cats and kittens, Natural Chicken Formula	*Salmonella* spp.		Feline's pride	FDA (2010a)	RMBD
30‐07‐2010	Iams, Eukanuba	Dry Pet Food Product	*Salmonella* spp.		The Procter and Gamble Company	FDA (2010g)	Dry
13‐08‐2010	Merrick	Beef Filet Squares for Dogs and Texas Hold'ems	*Salmonella* spp.		Merrick Petcare Inc.	FDA (2010c)	Treat
03‐09‐2010	Hartz Naturals	Hartz Naturals Real Beef Treats for Dog	*Salmonella* spp.		Hartz Mountain Corporation	FDA (2010i)	Treat
28‐01‐2011	Merrick JR Texas Taffy Pet Treats	Pet Treats	*Salmonella* spp.		Merrick Petcare Inc.	FDA (2011f)	Treat
08‐03‐2011	Jones Natural Chews, Blain's Farm and Fleet, Country Butcher Dog Chews	Pig ears	*Salmonella* spp.		Jones, Natural Chews Co.	FDA (2011d)	Treat
03‐05‐2011	Pig Ears for Pet Treats	Pig Ears	*Salmonella* spp.		Keys Manufacturing Company, Inc.	FDA (2011e)	Treat
17‐05‐2011	Digger's Natural Chews	Pig Ear Pet Treats	*Salmonella* spp.		Boss Pet Products, Inc	FDA (2011b)	Treat
17‐05‐2011	Prime Time, KC Beefhide	Pig ears	*Salmonella* spp.		Blackman Industries, Inc.	FDA (2011a)	Treat
28‐05‐2011	Primal Pet foods	Feline Chicken & Salmon Formula	*Salmonella* spp.		Primal pet foods	FDA (2011h)	RMBD
03‐06‐2011	Bravo!	Oven roasted Pig Ears Product	*Salmonella* spp.		Bravo!	FDA (2011c)	Treat
27‐06‐2011	Cat Chow, Friskies	Dry Cat Food	*Salmonella* spp.		Nestle Purina PetCare Company	FDA (2011i)	Dry
29‐07‐2011	Purina One	Dry Cat food	*Salmonella* spp.		Nestle Purina PetCare Company	FDA (2011g)	Dry
26‐04‐2012	Chiken Soup for the Pet Lover's Soul	Dry Dog Food	*Salmonella* spp.		Diamond Pet Foods	FDA (2012f)	Dry
30‐04‐2012	Diamond	Puppy Formula Dry Dog Food	*Salmonella* spp.		Diamond Pet Foods	FDA (2012g)	Dry
04‐05‐2012	Natural Balance	Dry pet food formulas	*Salmonella* spp.		Natural Balance Pet Food, Inc. (manufactured by Diamond Pet Foods at their Gaston, SC facility)	FDA (2012j)	Dry
04‐05‐2012	Apex	Dry Dog Food	*Salmonella* spp.		Apex Pet Foods	FDA (2012a)	Dry
05‐05‐2012	Diamond, Chicken Soup for the Pet Lover's Soul, Country Value, Daimond Nuturals, Premium Edge, Professional, 4Health, Taste of the Wild	Dry Dog Food	*Salmonella* spp.		Diamond Pet Foods	FDA (2012d)	Dry
05‐05‐2012	Canidae	Dry Pet Food Formulas	*Salmonella* spp.		Canidae Pet Foods (manufactured by Diamond Pet Foods at their Gaston, SC facility)	FDA (2012b)	Dry
07‐05‐2012	Wellness	Complete Health Super5Mix®	*Salmonella* spp.		WellPet LLC (manufactured by Diamond Pet Foods at their Gaston, SC facility)	FDA (2012m)	Dry
08‐05‐2012	Solid Gold WolfKing, Solid Gold WolfCub	Dry Dog Food (for puppy and adult dog)	*Salmonella* spp.		Solid Gold Health Products Fod Pets, Inc.	FDA (2012l)	Dry
21‐05‐2012	Diamond Naturals	Small Breed Adult Dog Lamb & Rice Formula dry dog foods	*Salmonella* Infantis*	49 human illnesses (47 in 20 US states and 2 in Canada) reported	Diamond Pet Foods	FDA (2012e); CDC (2012b)	Dry
21‐09‐2012	Boots & Barkley	American Beef Bully sticks	*Salmonella* spp.		Kasel Associated Industries	FDA (2012i)	Treat
02‐10‐2012	Nature's Deli	Chicken Jerky Dog Treats	*Salmonella* spp.		Kasel Associated Industries	FDA (2012h)	Treat
13‐10‐2012	Nature's Recipe	Oven Baked Biscuits with Real Chicken	*Salmonella* spp.		Del Monte Foods	FDA (2012k)	Treat
01‐11‐2012	Charlee Bear	Dog Food‐Protein Crunch Bars (chicken recipe with carrrots/ chicken recipe with sweet potatoes)	*Salmonella* spp.		Charlee Bear Products	FDA (2012c)	Treat
19‐02‐2013	Boots & Barkley, BIXBI, Nature's Deli, Colorado Naturals, Petco, and Best Bully Stick items	Dog Treats	*Salmonella* spp.		Kasel Associated Industries	FDA (2013g)	Treat
20‐02‐2013	NutriPet, Nutri‐Vet	Chicken Jerky Products	*Salmonella* spp.		Nutri‐Vet, LLC	FDA (2013n)	Treat
21‐02‐2013	Verve®, Zeal®, Thrive®	Dry Pet food products	*Salmonella* spp.		The Honest Kitchen	FDA (2013q)	Dry
21‐02‐2013	Boots & Barkley, Nature's Deli, more	Dog Treats	*Salmonella* spp.		Kasel Associated Industries	FDA (2013h)	Treat
06‐03‐2013	Jones Natural Chews Co	Woofers (beef patties) dog Treats	*Salmonella* spp.		Jones Natural Chews Co	FDA (2013f)	Treat
07‐03‐2013	Steve's Real Food	Frozen Pet Food‐Turducken Canine Diet	*Salmonella* spp.		Steve's Real Food	FDA (2013p)	RMBD
07‐03‐2013	Strippin' Chicks	Pet Treats	*Salmonella* spp.		Diggin' Your Dog	FDA (2013e)	Treat
13‐03‐2013	Bravo!	Raw Food Diet Chicken Blend for Dogs and Cats	*Salmonella* spp.		Bravo!	FDA (2013c)	RMBD
18‐03‐2013	California, Natural, EVO, Heathwise, Innova	Dry Pet Food	*Salmonella* spp.		Natura Pet Products	FDA (2013l)	Dry
29‐03‐2013	California Natural, EVO, Health Wise, Innova, Karma	Dry Pet food and Pet Treat	*Salmonella* spp.		Natura Pet Products	FDA (2013j)	Treat and Dry
03‐04‐2013	Bravo!	Raw Diet Frozen Foods for Dogs and Cats (Chicken balance, Chicken blend, Beef blend)	*Salmonella* spp.		Bravo!	FDA (2013b)	RMBD
04‐11‐2013	Bailey's choice	Pet Treats	*Salmonella* spp.		Bailey's Choice, LLC	FDA (2013a)	Treat
19‐04‐2013	California Natural, EVO, Health Wise, Innova, Karma	Dry Pet food and Pet Treat	*Salmonella* spp.		Natura Pet Products	FDA (2013i)	Treat and Dry
18‐06‐2013	Innova, EVO, California Natural, Healthwise, Karma, Mother Nature	Dry Pet food, biscuits/bar/Treats	*Salmonella* spp.		Natura Pet Products	FDA (2013k)	Treat and Dry
30‐08‐2013	Purina ONE beyOnd	Our White Meat Chicken and Whole Barley Recipe Adult Dry Dog Food	*Salmonella* spp.		Nestle Purina PetCare Company	FDA (2013m)	Dry
14‐08‐2013	Iams, Eukanuba	Dry Dog and Cat Foods	*Salmonella* spp.		Proctor & Gamble	FDA (2013o)	Dry
2013	Not specified	Chicken Jerky Pet Treats	*Salmonella* Typhimurium*	43 illnesses in New Hampshire and 16 were hospitalized	New Hampshire‐ based company	Cavallo et al. (2015)	Treat
25‐01‐2014	Red Flannel®	Dry Cat Food	*Salmonella* spp.		PMI Nutrition, LLC (manufactured by a third‐party manufacturer)	FDA (2014f)	Dry
05‐02‐2014	Hubbard Life, Joy, QC Plus	Dry Dog and Cat Foods	*Salmonella* spp.		Pro‐Pet LLC	FDA (2014g)	Dry
08‐04‐2014	Abady	Highest Quality Maintenance & Growth Formula for Cats	*Salmonella* spp.		Robert Abady Dog Food Co., LLC	FDA (2014h)	Dry
27‐05‐2014	Pet Center, Inc.	Lamb Crunchys Dog Treats	*Salmonella* spp.		Pet Center, Inc.	FDA (2014e)	Treat
02‐06‐2014	Hill's Science Diet	Dry dog Food	*Salmonella* spp.		Hill's Pet Nutrition, Inc.	FDA (2014c)	Dry
26‐09‐2014	Bravo!	Chicken and Turkey Blend	*Salmonella* spp.		Bravo!	FDA (2014b)	RMBD
24‐12‐2014	Barkworthies	Chicken Vittles Dog Chews	*Salmonella* spp.		Barkworthies	FDA (2014a)	Treat
31‐12‐2014	Jump Your bones	Roo Bites (Cubes) Pet Treats	*Salmonella* spp.		Jump Your Bones, Inc.	FDA (2014d)	Treat
16‐01‐2015	Oma's Pride	Purr‐Complete Feline Poultry Meal	*Salmonella* spp.		Oma's Pride	FDA (2015k)	RMBD
30‐01‐2015	Big Bark	Beef Jerky Treats for Dogs	*Salmonella* spp.		Grill‐Phoria LLC	FDA (2015e)	Treat
11‐02‐2015	Nutrisca	Chicken and Chick Pea Recipe Dry Dog Food	*Salmonella* spp.		Tuffy's Pet Foods, Inc.	FDA (2015n)	Dry
22‐04‐2015	Nylabone	Puppy Starter Kit dog chews	*Salmonella* spp.		TFH Publications, Inc./Nylabone Products	FDA (2015l)	Treat
15‐05‐2015	OC Raw	Turkey & Produce Raw Frozen Canine Formulation	*Salmonella* spp.		OC Raw Dog	FDA (2015i)	RMBD
19‐06‐2015	Boulder Dog Food Company	Chicken Sprinkles	*Salmonella* spp.		Boulder Dog Food Company, LLC	FDA (2015b)	Food Enhancer
02‐07‐2015	Boulder Dog Food Company	Turkey Sprinkles	*Salmonella* spp.		Boulder Dog Food Company, LLC.	FDA (2015a)	Food Enhancer
14‐07‐2015	I and Love and YOU	Cow‐Boom! Strips Beef Gullet Dog Chews	*Salmonella* spp.		NatPets LLC d/b/a “I and Love and You”	FDA (2015f)	Treat
20‐07‐2015	The Natural Dog Company	12" Tremenda Sticks Pet Chews	*Salmonella* spp.		The Natural Dog Company, Inc.	FDA (2015m)	Treat
23‐07‐2015	Bravo	Raw Chicken Pet Food for Dogs and Cats	*Salmonella* spp. Poly A		Bravo Pet Foods	FDA (2015d)	RMBD
24‐07‐2015	Instinct®	Raw Chicken Formula for dogs	*Salmonella* spp.		Nature's Variety	FDA (2015h)	RMBD
25‐09‐2015	OC Raw	Chicken, Fish & Produce Raw Frozen Canine Formulations	*Salmonella* spp.		OC Raw Dog	FDA (2015j)	RMBD
02‐10‐2015	K‐9 Kraving Dog Food	Chicken Patties Dog food	*Salmonella* and *Listeria monocytogenes*		K‐9 Kraving Dog Food	FDA (2015g)	RMBD
10‐12‐2015	Bravo	Chicken Blend diet for dogs & cats	*Salmonella* spp.		Bravo Pet Foods	FDA (2015c)	RMBD
04‐01‐2016	Big Dog Natural (BDN)	Raw Dehydrated dog food (Chicken Supreme)	*Salmonella* spp.		Big Dog Natural	FDA (2016a)	RMBD
26‐03‐2016	Smallbatch Pets	Frozen Dog Duckbatch Sliders	*Salmonella* and *Listeria monocytogenes*		Smallbatch Pets Inc.	FDA (2016d)	RMBD
23‐06‐2016	Rad Cat	Raw Cat Food	*Salmonella* and/or *Listeria monocytogenes*		Radagast Pet Food, Inc.	FDA (2016c)	RMBD
08‐12‐2016	Blue Ridge Beef	Frozen Pet Food (beef for dogs and kitten grind)	*Salmonella* and/or *Listeria monocytogenes*		Blue Ridge Beef	FDA (2016b)	RMBD
20‐03‐2017	Barnsdale Farms®, HoundsTooth® and Mac's Choice®	Pig Ears	*Salmonella* spp.		EuroCan Manufacturing	FDA (2017a)	Treat
05‐05‐2017	Smallbatch Pets	Chicken Blend for dogs and cats	*Salmonella* spp.		Smallbatch Pets Inc.	FDA (2017c)	RMBD
14‐06‐2017	Loving Pets	Dog Treats	*Salmonella* spp.		Loving Pets	FDA (2017b)	Treat
08‐02‐2018	Raws for Paws	Ground turkey pet food	*Salmonella* Reading (MDR)*	Reported Cases: 358 Hospitalizations: 133 Deaths: 1 (4 of the 200 ill people interviewed became sick after pets in their home ate raw ground turkey pet food)	Christofersen Meats Company, Inc., dba Swanson Meats	FDA (2018c); CDC (2018)	RMBD
02‐03‐2018	Blue Ridge Beef	Kitten Grind raw pet food	*Salmonella* and *Listeria monocytogenes*		Blue Ridge Beef	FDA (2018a)	RMBD
02‐03‐2018	Steve's Real Foods	Raw Frozen Dog Food Turkey Canine Recipe	*Salmonella* spp.		Steve's Real Foods	FDA (2018e)	RMBD
26‐03‐2018	Blue Ridge Beef	Complete Raw Pet Food	*Salmonella* and *Listeria monocytogenes*		Blue Ridge Beef	FDA (2018b)	RMBD
21‐12‐2018	Columbia River Natural Pet Foods	Frozen meat product for dogs and cats	*Salmonella* and *Listeria monocytogenes*		Columbia River Natural Pet Foods	FDA (2018d)	RMBD
28‐01‐2019	Woody's Pet Food Deli	Raw Free Range Turkey	*Salmonella* Reading (MDR)*	Reported Cases: 358 Hospitalizations: 133 Deaths: 1 (4 of the 200 ill people interviewed became sick after pets in their home ate raw ground turkey pet food)	Woody's Pet Food Deli	CDC (2018)	RMBD
03‐07‐2019	Multiple brands	Pig ears	Cerro, Derby, London, Infantis, Newport, Rissen, and I 4,[5],12:i:‐* (MDR)	154 cases in 34 states; 35 Hospitalizations. 27 cases were < 5 years old	Multiple companies	FDA (2019a)	Treat
18‐11‐2019	Quest	Beef Cat Food	*Salmonella* spp.		Go Raw, LLC	FDA (2019c)	RMBD
03/03/2021	Bravo Packing, Inc.	Frozen Raw Pet Food (Ground Beef and Performance Dog)	*Salmonella* and *Listeria monocytogenes*		Bravo Packing, Inc.	FDA (2021a); FDA (2019b)	RMBD
16‐03‐2021	Bravo Packing, Inc.	Frozen Raw Pet Food (all pet food and bones in all package sizes)	*Salmonella* and *Listeria monocytogenes*		Bravo Packing, Inc.	FDA (2021b)	RMBD
27‐03‐2021	Multiple brands (CanineX, Earthborn Holistic, Venture, Unrefined, Sportmix Wholesomes, Pro Pac, Pro Pac Ultimates, Sportstrail, Sportmix and Meridian)	Dog and Cat Food	*Salmonella* spp.		Midwestern Pet Foods	FDA (2021e)	Dry
12‐04‐2021	Meow Mix®	Original Choice Dry Cat Food	*Salmonella* spp.		J. M. Smucker Co.	FDA (2021h)	Dry
20‐05‐2021	Natural Balance	Limited Ingredient Diets (LID) Green Pea & Chicken Dry Cat Food	*Salmonella* spp.		Natural Balance Pet Foods, Inc.	FDA (2021f)	Dry
03‐06‐2021	Sportsman's Pride, Sprout Sporting, Intimidator, FRM Gold Select	Dog Food Products	*Salmonella* spp.		Sunshine Mills, Inc.	FDA (2021g)	Dry
13‐06‐2021	Freshpet	Select Small Dog Bite Size Beef & Egg Recipe Dog Food	*Salmonella* spp.		Freshpet Inc.	FDA (2021c)	Cooked wet pet food
26‐08‐2021	Top Quality Dog Food.com	Beef HVM	*Salmonella* and *Listeria monocytogenes*		Top Quality Dog Food, LLC	FDA (2021i)	RMBD
23‐12‐2021	Woody's Pet Food Deli	Raw Cornish Hen pet food “With Supplements”	*Salmonella* spp.		Woody's Pet Food Deli	FDA (2021d)	RMBD
18‐02‐2022	Multiple brands	Animal (pet) food (including human food, medical devices and drug products)	*Salmonella* spp. (associated with the presence of rodents and rodent activity)		Family Dollar, Inc.	FDA (2022a)	Undetermined
18‐06‐2022	Freshpet	Freshpet Select Fresh From the Kitchen Home Cooked Chicken Recipe	*Salmonella* spp.		Freshpet Inc.	FDA (2022b)	Cooked wet pet food
12‐07‐2022	Beg & Barker, Billo's Best Friend, and Green Coast Pets	Chicken dog Treats	*Salmonella* spp.		Stormberg Foods	FDA (2022c)	Treat
16‐12‐2022	HEB Texas Pets	Indoor Complete Dry Cat Food	*Salmonella* spp.		TFP Nutrition	FDA (2022d)	Dry
23‐08‐2023	Multiple brands	Animal (pet) food (including human food, medical devices and drug products)	*Salmonella* spp. (associated with the presence of rodents and rodent activity)		Inmar Supply Chain Solutions	FDA (2023c)	Undetermined
21‐10‐2023	Retriever	Mini Chunk Chicken Recipe Dry Dog Food	*Salmonella* spp.		Texas Farm Products Company dba TFP Nutrition	FDA (2023d)	Dry
27‐10‐2023	Blue Ridge Beef	Breeders Choice Raw Pet Food	*Salmonella* spp.		Blue Ridge Beef	FDA (2023a)	RMBD
09‐11‐2023	Victor, Eagle Mountain, Wayne Feeds and two varieties of Member's Mark pet foods	Dog and Cat Food	*Salmonella* Kiambu*	7 illnesses and 1 hospitalization in 7 states. 6 of the 7 cases were < 1 year old	Mid America Pet Food	CDC (2024c)	Dry
16‐11‐2023	Multiple brands	Dry Dog and Cat Food (including Catfish Food)	*Salmonella* spp.		TFP Nutrition	FDA (2023e)	Dry
03‐01‐2024	Blue Ridge Beef	Kitten Grind, Kitten Mix, and Puppy Mix	*Salmonella* and *Listeria monocytogenes*		Blue Ridge Beef	FDA (2024a)	RMBD
28‐03‐2024	Not mentioned	Raw Pet Food	*Salmonella* I 4,[5],12:i:‐ (XDR) *	44 laboratory‐confirmed cases in six provinces in Canada, 13 hospitalizations. 43% of the cases were < 5 years old	Not mentioned	Public Health Agency of Canada (2024)	RMBD
23‐09‐2024	Answers Pet Food products	Raw Beef Detailed Formula for Dogs, Raw Beef Straight Formula for Dogs, Straight Chicken Formula for Dogs	*Salmonella* and *Listeria monocytogenes*		Lystn LLC.	FDA (2024e)	RMBD

*‐Linked to human outbreaks

†Date is presented in this format: day/month/ year. In other recalls/outbreaks, only the year is provided.

MDR‐Multi‐drug resistant, XDR‐ Extensive‐drug resistant

Among human cases of *Salmonella* outbreaks associated with pet food, pig ear dog treats associated with a 1999 outbreak in Canada are noteworthy. Thirty dog owners, many of whom were children, handled and/or fed the treats to their dogs and tested positive for *S*. Infantis. Follow‐up studies after this outbreak revealed that pig ears were frequently associated with *Salmonella* (Clark et al., 2001; White et al., 2003). Subsequently, in 2002 in Calgary, Canada, pet treats sold by a Texas‐based company were associated with outbreaks of *S*. Newport PT 14 in humans. A total of five human cases were reported, including one 1‐month‐old infant (Finley et al., 2006). The *Salmonella* serovar in this outbreak was traced back to commercial pet treats, and all the households identified as positive were reported to have fed the same sourced pet treats—dried‐up beef patties. In this outbreak, it was the pet owners who got infected and not the pet animals, as their fecal samples showed negative results (Pitout et al., 2003), highlighting the risk associated with the handling of contaminated pet food.

A 2005 human outbreak of *S*. Thompson was linked back to frozen raw beef and salmon in pet treats for cats and dogs (Finley et al., 2006). This led to a total of nine human‐culture–confirmed *Salmonella* cases in Washington state and Western Canada. Upon investigation, it was found that the dehydration temperature applied to the treats was not high enough to kill the pathogen, and no other pathogen‐killing steps were involved. All the infected people were reported to have handled pet food from a common source, and the oldest person infected was aged 81. Similarly, a notorious pet food‐linked *Salmonella* outbreak in humans in 2007–2008 was associated with dry dog and cat food originating from one of the Pennsylvania plants (Deasy et al., 2008). A total of 79 human cases in 21 states were reported positive for the *S*. Schwarzengrund. Upon investigation, the *Salmonella* serovar was isolated from one of the processing rooms in the plant. Based on the available data on the infected people, 39% of them were 1 year of age or younger. A dry dog food linked to the human *S*. Infantis outbreak in 2012 was reported to be associated with a manufacturing plant based in South Carolina. The outbreak caused 49 human illnesses: 47 in 20 U.S. states and two in Canada (FDA, 2012e), and among 24 infected people with available information, 10 (42%) were hospitalized. However, the age‐based patient information was not available.

In another pet treat associated with *Salmonella* outbreaks in humans from 2013, locally made jerky pet treats in New Hampshire led to a human outbreak that caused 43 illnesses with 16 (37%) hospitalizations (Cavallo et al., 2015). Among the infected patients, 69% were exposed to contaminated pet treats, and 95% of them claimed they were exposed to treat‐fed dogs. Upon investigation, the manufacturing site of the pet treats revealed inadequate processing and improper sanitary measures during production and packaging. Additionally, 78% (7/9) of environmental samples of the site were positive for the outbreak strain. Similarly, in 2018, contaminated raw ground turkey pet food was associated with human cases of *Salmonella* (FDA, 2018c). The outbreak led to two human illness cases. However, further details about the infected patients were not provided. Further testing of the suspected turkey pet foods revealed the presence of the *Salmonella* spp.

In yet another pet treat‐associated human *Salmonella* outbreak, pig ear pet treats from multiple brands were linked to a massive *Salmonella* outbreak in 2019 that led to 154 illnesses with 35 (26%) hospitalizations in 34 states (FDA, 2019a). The affected people ranged from 1 to 90 years old. Twenty‐seven (19%) of the illnesses were among children younger than five years of age. *Salmonella* serovars Cerro, Derby, London, Infantis, Newport, Rissen, and I 4,[5],12:i:‐ were associated with the outbreaks. Notably, multiple MDR serovars were isolated from this outbreak; however, the details of the MDR serovars were not provided (CDC, 2019a). The biggest and most severe *Salmonella* outbreak associated with pet food to date was reported in 2017–2019, which was linked to raw turkey products (CDC, 2018). A total of 358 people were infected with the outbreak strain of *S*. Reading in 42 states, causing 133 hospitalizations (44%) and one death. The age of the infected people ranged from 1–101 years, with 42 being the median. Four out of 200 people interviewed reported getting sick after feeding raw ground turkey to their pets, while the majority were reported to have been eating or preparing turkey. Out of the total isolates analyzed, 64% (314/587) were MDR. Upon investigation, the outbreak serovar was isolated from raw turkey products, raw turkey pet food, and live turkeys.

Similarly, in 2023, dry pet food was associated with an outbreak of *S*. Kiambu (FDA, 2023b). As per the latest update, this outbreak led to 7 illnesses and 1 hospitalization. However, the number of sick people in this outbreak was expected to be much higher. The alarming report from this outbreak was that 86% of the infected people were 1 year of age or younger, and the remaining 14% were 65 years and older. The most recent raw pet food‐linked MDR *Salmonella* outbreak in humans was associated with raw pet food and contact with cattle (Public Health Agency of Canada, 2024). The outbreak led to 44 illnesses and 13 hospitalizations, with the infected people ranging from 1 year to 91 years of age. A significant number of the cases (43%) were in children 5 years of age or younger. Another concerning finding from this outbreak was that the *Salmonella* strain I 4,[5],12:i:‐, associated with this outbreak, was extensively drug‐resistant, including to those commonly used human clinical medicine.

**FIGURE 2:**
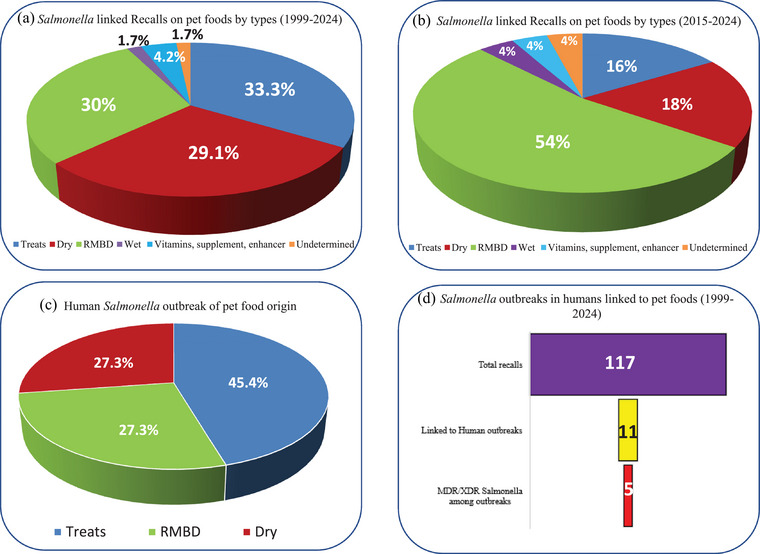
*Salmonella*‐linked outbreaks and recalls of pet foods.

*Salmonella*‐linked pet food recalls between 1999 and June 2024 were categorized by the types of food: 33.3% were linked to pet treats, 29.1% were to dry pet food, 30% were to RMBD, 4.2% were to vitamins and supplements, and 1.7 % were to wet food (Figure 2a). However, when such recalls were analyzed between 2015 and 2024, 54% of the recalls were associated with RMBD, compared with 16% with pet treats, 18% with dry, and 4 % with supplements (Figure 2b). There seems to be a trend as to the type of pet food in human *Salmonella* outbreaks, which may be linked to the growing popularity of RMBD. Similarly, out of the total human *Salmonella* outbreaks associated with pet food types, 45.5% (5/11) were linked to pet treats, 27.3 % (3/11) were linked to RMBD, and 27.3 % (3/11) were linked to dry pet foods (Figure 2c). This could be correlated to the fact that RMBD and pet treats are minimally treated to mitigate pathogens. Finally, out of the total *Salmonella*‐associated recalls in pet foods between 1999 and 2024, 9.5% of them were linked to human *Salmonella* outbreaks. What was more concerning was that 45.5% of those human outbreaks were linked to MDR *Salmonella* (Figure 2d), with one being extensively MDR (XDR). The next section defines the terms MDR and XDR. From 2021 to June 2024 alone, there were 21 *Salmonella*‐linked pet food recalls in the United States, out of which 8 were linked to raw pet foods (Table 2). Another notable observation from the above‐described outbreaks was that children and the elderly are the largest groups of people infected, highlighting the vulnerability of kids and elderly in pet food‐related outbreaks.

The U.S. FDA follows a systematic process to handle pet food recalls. The FDA investigates reports of contamination, illness, and other safety concerns related to pet food in collaboration with state regulatory agencies and other stakeholders. If the FDA determines that a pet food product potentially contains pathogens, it will work closely with the manufacturer and may request the manufacturer to recall the product voluntarily. If a manufacturer refuses to initiate a voluntary recall, the FDA has the authority to order a mandatory recall under the Food Safety Modernization Act (FSMA). FDA oversees inspections of pet food manufacturers and suppliers of ingredients (excluding those regulated by the USDA FSIS) and conducts investigations in response to consumer complaints. However, it is often frowned upon amongst pet owners and pet food customers that the enforcement of these regulations is not consistently rigorous, and the regulatory body, the FDA, does a risk‐based inspection. The CDC collaborates with the FDA and USDA when contaminated pet food causes human illness solely as a public health agency without regulatory oversight.

## 7 MDR *SALMONELLA* IN PET FOODS

The use of antibiotics in animal agriculture, including the production of ingredients used in pet foods, can contribute to the development of antibiotic‐resistant strains of *Salmonella*. MDR nontyphoidal *Salmonella* was categorized under the ‘serious threat’ category by the U.S. CDC in 2019 (CDC, 2019b). The use of antimicrobials in animal and pet food might have been linked to the emergence of MDR strains of bacteria. Multidrug resistance is defined by the lack of susceptibility of a pathogen to at least one antimicrobial agent in three or more distinct categories of antimicrobials. Whereas extensively drug‐resistant (XDR) refers to microorganisms that exhibit susceptibility restricted to no more than two categories of antimicrobial agents (McDermott et al., 2018; Magiorakos et al., 2012).

Many prevalence studies and pet food recalls have documented MDR *Salmonella* in pet foods. The study by Wong et al. (2007), as highlighted in Table 2, reported that *S*. London (ampicillin and gentamicin) and *S*. Infantis (nalidixic acid and streptomycin) isolated from pet chews were resistant to human antibiotics. Similarly, Finley et al. (2008) reported that several *Salmonella* isolates from raw pet foods from Mississauga, Calgary, and Guelph in Canada were resistant to 1 to 5 human antibiotics. In 2019, *Salmonella* serovar 4,5,12:i:‐ and Thompson isolated from dog treats were resistant to one or more antibiotics (Yukawa et al., 2019). A 2021 study by Degi et al. (2021), who isolated 16 *Salmonella* serovars from cat feces reported all serovars [Typhimurium (*n* = 4; 25%), Enteritidis (*n* = 9; 56.3%), and Kentucky (*n* = 3; 18.8%)] as MDR with strong resistance towards cefazolin, cefepime, ceftazidime, and ceftriaxone. Further, resistance against trimethoprim/sulfamethoxazole (11/16; 68.8%), ampicillin (10/16; 62.5%), ampicillin/sulbactam (9/16; 56.3%), gentamicin (9/16; 56.3%), nitrofurantoin (8/16; 50.0%), and amikacin (5/16; 31.3%) were also observed. In another set of studies by the same team, MDR *Salmonella* was isolated from raw pet foods in Japan, where *S*. Infantis was reported to be resistant to streptomycin, kanamycin, tetracycline, and trimethoprim, and *S*. Typhimurium to nalidixic acid, ciprofloxacin, and chloramphenicol (Yukawa et al., 2022). Despite several cases of isolated MDR *Salmonella* from dog food, the following section focuses on the pet food associated with MDR *Salmonella*, which was part of human outbreaks.

Out of the total 212 isolates (110 from humans and 102 from pig ear pet treats), 164 (77%) were reported to possess some MDR in the 2019 salmonellosis outbreak that sickened 154 people. The isolates were resistant to many commonly used antibiotics such as amoxicillin‐clavulanic acid (<1%), ampicillin (53%), azithromycin (<1%), cefoxitin (<1%), ceftriaxone (<1%), chloramphenicol (33%), ciprofloxacin (50%), fosfomycin (2%), gentamicin (27%), kanamycin (2%), nalidixic acid (26%), streptomycin (33%), sulfisoxazole (30%), tetracycline (58%), and trimethoprim‐sulfamethoxazole (27%) (CDC, 2019c). In another major MDR *Salmonella* outbreak in humans in the same year, raw products, raw turkey pet food, and live turkey were the source of *Salmonella* (CDC, 2019d). Upon whole‐genome sequencing (WGS) of 487 *S*. Reading isolates, 314 isolates (64%) were reported to be resistant to ampicillin (52% of all 487 isolates), streptomycin (32%), sulfamethoxazole (31%), tetracycline (32%), kanamycin (3.4%), gentamicin (0.6%), nalidixic acid (0.4%), ciprofloxacin (0.4%), trimethoprim‐sulfamethoxazole (0.4%), and fosfomycin (0.2%).

Similarly, an interesting finding from Charite University Hospital, Germany, reported a possible passing of MDR organisms from dogs and cats to their owners (Hackmann et al., 2023). The case–control study isolated MDR *Salmonella* from pet owners and linked it to their dogs and cats. In a separate study, commercial RMBDs in Japan were positive for MDR *S*. Infantis (*n* = 3), *S*. Typhimurium (*n* = 1), and *S*. Schwarzengrund (*n* = 1). (Yukawa et al., 2022). All 5 isolates were susceptible to ampicillin, cefazolin, cefotaxime, and gentamycin; 2 isolates were resistant to >1 antibiotic; 1 *S*. Infantis was resistant to streptomycin, kanamycin, tetracycline, and trimethoprim, whereas the *S*. Typhimurium isolate was resistant to nalidixic acid, ciprofloxacin, and chloramphenicol and *S*. Schwarzengrund isolate was resistant to tetracycline.

One of the latest MDR *Salmonella* isolated from human outbreaks associated with pet foods was linked to raw pet food and contact with cattle (Public Health Agency of Canada, 2024). *Salmonella* I 4,[5],12:i: isolated from the outbreak, was extensively drug‐resistant and was resistant to most of the commonly used antibiotics, such as ceftriaxone, azithromycin, trimethoprim/sulfamethoxazole, ampicillin, and ciprofloxacin. The strain was also resistant to older antibiotic drugs such as aminoglycosides, chloramphenicol, and tetracycline.

## 8 *SALMONELLA* RISK MITIGATION STRATEGIES IN THE PET FOOD INDUSTRY

Different intervention strategies are often applied to mitigate *Salmonella* contamination and encompass a range of preventive measures across the food production continuum. Aside from the conventional thermal lethality step during the extrusion processing of pet food, nonthermal processing and chemical and biological interventions are often used as integrated strategies to form a comprehensive approach to mitigating *Salmonella* contamination in pet foods. Table 3 provides a detailed list of the most common intervention strategies in different pet food types to control *Salmonella*.

**TABLE 3:** Reported interventions used to mitigate *Salmonella* in pet foods

No.	Interventions	Pet food substrate	Target pathogen(s)	*Salmonella* reduction	References
**A**.	**Physical Interventions**				
1	High pressure processing (HPP)	Raw beef pet food	*E. coli* ATCC BAA 1427‐31 (as surrogate for *Salmonella)*	4.9‐6.2 log	Hasty et al. (2018)
		Chicken‐based Raw pet food	*Salmonella, E. coli *and *Listeria monocytogenes*	<0.5 log to ∼4.9 log	Serra‐Castello et al. (2022a)
		Chicken‐based Raw pet food intended for dogs	*Salmonella* Derby CTC1022, Typhimurium GN0085 and Enteritidis GN0082	0.76 to >9 log	Serra‐Castello et al. (2022b)
		Chicken‐based Raw pet food intended for cats	*Salmonella* Derby CTC1022, Typhimurium GN0085 and Enteritidis GN0082	1‐9 log	Serra‐Castello et al. (2023)
		Raw meat‐based pet food diet (RMBD), Bones and raw pet food (BARF)	*Salmonella, E. coli* and *Listeria monocytogenes*	NA; reported absence of *Salmonella* until Day 30	Neshovska et al. (2023)
		Pet food formulated with raw meat (chicken, beef and lamb)	*Salmonella, E. coli *(STEC), and* Listeria monocytogenes*	2.24 log to >5 log	Lee et al. (2023)
2	Cold plasma treatment	Freeze‐dried pet food treats	*Salmonella* Typhimurium and Senftenberg	3.03 log CFU/cm^2^ (maximum at Day 0), >4.5 log (after 7 days)	Yadav & Roopesh (2020)
3	Irradiation	Horse meat and kangaroo meat for use in pet food	*Salmonella* (Typhimurium phage type 14, Senftenberg 1502, Good, Oranienburg, Anatum, Minnesota)	at least 5 log	Ley et al. (1970)
		Commercial Semi‐moist pet food	*E. coli* O157:H7 (STEC) and *Salmonella* Typhimurium	Not indicated	Sethukali et al. (2023)
4	Pulsed light emitting diode (LED)	Dry pet food pellets	*Salmonella* Typhimurium (ATCC 13311), *S*. Senftenberg (ATCC 43845), *Salmonella* FUA1946, FUA1934, FUA1955, and *E. coli* AW1.7	0.79‐1.76 log CFU/g	Prasad et al. (2019)
		Dry pet food pellets	*Salmonella* Typhimurium ATCC13311 and Senftenberg ATCC43845	0.33‐2.26 log CFU/g	Subedi & Roopesh (2020)
**B**.	**Biological Interventions**				
1	Bacteriophage	Dry pet food (kibble)	*Salmonella* Enteritidis (ATCC 4931), Montevideo (ATCC 8387), Senftenberg (ATCC 8400), and Typhimurium (ATCC 13311)	0.75‐2.05 log reduction MPN/g (in comparison to the negative control)	Heyse et al. (2015)
		Raw pet food ingredients (chicken, tuna, turkey, cantaloupe, and lettuce)	*Salmonella* Enteritidis (ATCC 13076), Typhi (ATCC 6539), and Heidelberg (ATCC 8326)	0.4‐1.1 log CFU/g	Soffer et al. (2016)
**C. Chemical Additives**	**Chemical Interventions**				
1	Organic acids	Meat and bone meal (MBM) used in pet food	*Salmonella* (8 serovars), *E. coli*, *S. aureus, C. perfringens, S. agalactiae*, and *C. jejuni*	∼1 log	O'Bryan et al. (2015)
		Dry pet food kibble	*Salmonella* Enteritidis (ATCC 13076)	4.9‐7.1 log	Huss et al. (2017)
		Dry pet food kibble	*Salmonella* Enteritidis (ATCC 4931), Heidelberg (ATCC 8326), and Typhimurium (ATCC 14028), *E. coli* (STEC)	2.2 log (after 2 hrs)	Deliephan et al. (2023a)
		Raw meat‐based diet (RMBD) for dogs	*Salmonella* Enteritidis (ATCC13076), Heidelberg (ATCC 8326), Typhimurium (ATCC 14802)	1.19‐6.09 log	Kiprotich et al. (2023)
2	Combination of Sodium bisulfate (SBS) with acids	Rendered chicken fat used in pet food	*Salmonella* Typhimurium (ATCC 14028)	2.7 to > 5.5 log	Dhakal et al. (2019b)
		Chicken fat, canola oil, fish oil, lard, and tallow used to coat dry pet food kibbles	*Salmonella* Enteritidis (ATCC 4931), Heidelberg (ATCC 8326), and Typhimurium (ATCC 14028)	>7 log within 2 h (lowered below detection limit)	Dhakal & Aldrich (2023)
3	Ozone treatment	Vegetables used in raw meat‐based pet food diet	*Salmonella* Javiana (ATCC BAA1593), Newport (ATCC 6962) and Typhimurium (ATCC 14028), and *Listeria monocytogenes*	0.51‐ 4.67 log CFU/ml	Chandran et al. (2023)
4	Medium‐chain fatty acids	Dry pet food kibble	*Salmonella *Typhimurium (ATCC 14028)	>4.5 log after 5 h	Dhakal & Aldrich (2020)
5	Fish oil	Fish oil used in pet food, and dry pet food kibble	*Salmonella* Enteritidis (ATCC 4931), Heidelberg (ATCC 8326), and Typhimurium (ATCC 14028)	1.25 log (compared to control)	Dhakal & Aldrich (2022)
6	Plant‐derived antimicrobials/botanical extracts	Dry pet food kibble	*Salmonella* Schwarzengrund	at least ∼2 log CFU/g to 3.5 log CFU/g	Chen et al. (2019)
7	Cultured milk or cultured dextrose	Chicken livers (used as ingredient in RMBD)	*Salmonella* Braenderup, Enteritidis, Hadar, Heidelberg and Typhimurium	0.43‐0.95 log	Cancio et al. (2023)

### 8.1 Physical interventions

#### 8.1.1 High‐pressure processing

Raw pet food manufacturers aim to maintain the ‘raw‐like’ attributes in their raw pet foods. Although traditional thermal pasteurization technology may negatively affect sensory characteristics, flavors, and nutritional contents of food, nonthermal processing technologies like HPP have attracted widespread attention from food industry practitioners. The HPP ensures microbial safety without the addition of preservatives and allows processed food to maintain the natural flavors and nutritional value of the original food material (Daryaei & Balasubramanian, 2012). In HPP technology, food is hermetically sealed in a flexible container under a high pressure of 100–600 MPa applied at room temperature using a liquid (typically water) as the pressure transfer medium, subjecting the interior and surface of the food to even pressure to achieve pasteurization (Balasubramaniam et al., 2015). Because the food is in packaged form and does not directly contact the processing devices, it prevents postprocessing contamination of food. This is critical in pet food manufacturing because postprocessing contamination is considered a key source of pathogen entry in pet foods. The HPP technology is commonly used to reduce pathogen levels and extend the shelf life of various human foods such as ready‐to‐eat foods, meat products, juices and beverages, seafood, and vegetable products. HPP‐related research, particularly targeting pet foods, is limited (Serra‐Castello et al., 2022b). This could be partly because the food constituents used in pet foods are derived or diverted from human food manufacturing, and an intervention that is effective in human foods is, in general, also effective in pet foods. However, this technology is now increasingly adopted by pet food producers worldwide (Serra‐Castello et al., 2022a). In the pet food industry, frozen raw and freeze‐dried pet foods and treats are commonly HPP treated.

Hasty et al. (2018) studied the effectiveness of HPP (600 MPa for 8 min) in raw beef pet food inoculated with 7 logs of a *Salmonella* surrogate (*E. coli* ATCC BAA 1427–31) and incubated at −23°C. After 24 h and 5 days of HPP treatment, log reductions of 4.9 logs and 6.2 logs were reported on selective agar. Similarly, Serra‐Castello et al. (2022b) inoculated raw pet food consisting of chicken, vegetables, antioxidants, vitamins, and minerals with a three‐serovar cocktail of *Salmonella* spp. (Derby, Typhimurium, and Enteritidis). The frozen block of pet food was vacuum‐packed and subjected to HPP treatment. A maximum reduction of 9.33 log was observed at 750 MPa for 3.5 min at a pH of 6.09 ± 0.05. The same team investigated the effect of HPP against the same three serovars in raw pet food prepared from chicken, plant‐based ingredients, salmon, and spices according to the commercial recipe and stored at −20°C until use. A 9.08 log reduction of *Salmonella* was observed after both day 0 and day 14 from samples were stored at −18°C post‐HPP (750 MPa for 3.5 min) treatment (Serra‐Castello et al., 2023). Similarly, in another recent study by Lee et al. (2023), three different raw pet food formulations with different levels of meat, organ meat, bone, seeds, fruits, and vegetables were inoculated with a 7‐log cocktail of 6 strains of *Salmonella* (3 of them were pet food isolates), treated with 586 MPa for 1 to 4 min and were either stored at 4°C or frozen at −10°C to −18°C for 21 days. Beef formulations were able to maintain the inactivation level above 5 logs after 586 MPa/2 min treatment and 1 day after storage and were maintained until the duration of the study.

Not only does HPP act as point‐in‐time mitigation in pet foods, but this intervention is also known to extend the shelf life of pet foods. Neshovska et al. (2023) applied HPP (600 MPa for 3 min) to 210 raw pet food samples consisting of three different diet compositions, including animal and plant‐derived constituents, and evaluated shelf life and pathogen prevalence over time after incubating at refrigerated temperature. Samples were analyzed after 0, 15, and 30 days for *Salmonella*, *E. coli*, and aerobic bacteria. Although the aerobic plate count was above the acceptable range after 15 days, the *E. coli* and *Salmonella* were not detected until the study period ended, indicating that the HPP potentially extended the shelf life of raw pet foods. Different pathogens in raw pet foods tend to show different pressure resistance depending on species and strains. In general, *Salmonella* and *L. monocytogenes* strains displayed higher pressure resistance to HPP when compared with *E. coli* strains. The study also reported that the addition of lactic acid markedly enhanced the effectiveness of HPP against *L. monocytogenes* (Serra‐Castello et al., 2022a)

#### 8.1.2 Cold plasma treatment

Cold plasma is a relatively new processing technology that uses an ionized gas consisting of neutral molecules, electrons, and positive and negative ions. It inactivates microbes via UV radiation and produces reactive chemical products of the cold plasma ionization process, including ozone, charged particles, reactive oxygen species (ROS), reactive nitrogen species, and free radicals (Hertrich et al., 2017). In‐package dielectric barrier discharge is one of the methods to generate cold plasma inside a confined food package, known as atmospheric cold plasma treatment. The ROS and charged particles possess tremendous potential to injure and inactivate several microorganisms like bacteria, fungi, and spores (Yadav & Roopesh, 2020). Cold plasma has been demonstrated to effectively reduce pathogens in several commodities, including seeds, fruits, vegetables, and pet treats (Hertrich et al., 2017; Yadav & Roopesh, 2020). Yadav and Roopesh (2020) studied the effect of cold plasma in freeze‐dried pet food treats inoculated with *Salmonella*. The surface inoculation of 8.2 log CFU/cm^2^ *Salmonella* cocktail (Typhimurium and Senftenberg) was followed by modified atmospheric packaging and in‐package atmospheric cold plasma (APC) treatment. A 10 min APC followed by 7‐day storage at room temperature (21°C) successfully reduced *Salmonella* counts by 4.5 logs. However, to the knowledge of the authors, cold plasma technology is not being used in any commercial pet food industry as a pathogen mitigation method.

#### 8.1.3 Pulsed light treatment

Light‐emitting diodes (LEDs) are semiconductor diodes that use electroluminescence properties to produce light. High‐intensity light (in the ultraviolet wavelength range of 100–400 nm) pulses emitted from LEDs can reduce surface contamination in low‐moisture foods, including pet foods (Subedi & Roopesh, 2020). UV‐A light (320–400 nm) exposure causes bacterial cell death by generating ROS within the cell. Pulsed UV treatment is used for surface decontamination (Subedi & Roopesh, 2020) of fruits and vegetables, meat and poultry, and low‐moisture and high‐moisture foods. However, there has been limited consumer acceptance of the usage of pulsed UV LEDs. In a study by Subedi and Roopesh (2020), the application of 395 nm LED treatment and 395 nm LED combined with vibration and mild hot air (50°C) caused a 1.2 and 2.26 log reduction in *Salmonella* spp. (Typhimurium and Senftenberg) levels in dry pet food pellets. In a separate study by Prasad et al. (2019), dry pet food pellets with water activity of *ca*. 0.54 were inoculated with a five‐strain cocktail of *Salmonella* spp. (9 log CFU/mL) and air‐dried for 45–60 min. The pet food was treated at a 2 cm distance from the LED light source at spectra of 365 and 395 nm. The 395 nm LED treatment showed a significant *Salmonella* inactivation (1.76 log reduction) compared with 365 nm (0.79 log reduction). Similar to cold plasma, to our knowledge, pulse light treatment has not been used in any commercial pet food industry as a pathogen reduction step.

#### 8.1.4 Irradiation

Food irradiation is the process whereby foodstuffs are exposed to a source of ionizing radiation. The irradiation technology was approved in pet foods, pet treats (including pig ears), and chews in 2001 (American Veterinary Medical Association (AVMA), 2001). Pet foods are exposed to sources of ionizing radiation, which can cause chemical changes. The approval from the FDA was obtained after a petition was filed to control the risk of *Salmonella* in pet foods, which was identified as a potential threat to pet owners, especially children. The radiation sources, depending on dosage, either destroy or render pathogens incapable of reproduction. There are very limited studies available on the use of irradiation to mitigate pathogens in pet food. Unfortunately, irradiation in pet foods is not widely appreciated by pet owners, especially after the 2007–2008 reports from Australia, where as many as 87 cats developed neurological symptoms and were suspected to be due to the feeding of irradiated dry pet food. The vitamin A depletion due to gamma irradiation was suggested to be a possible cause of neurologic symptoms in the affected cats (Child et al., 2009). However, in a study by Zhu et al. (2012), the physiological effect of feeding irradiated pet foods to pet rats was studied and it reported that pet foods irradiated at 10, 15, and 25 kGy did not cause any abnormal physiological parameters as measured in terms of the general situation, food intake, food utilization rate, hematological parameters, biochemical parameters, viscera weight, histopathological reports, height, tail length, body temperature, heart rate, blood pressure, etc. when compared with the control groups. In one of the older studies published by Ley et al. (1970), raw frozen (−15°C) meat (horsemeat, kangaroo, and veal) intended for use as pet food was gamma irradiated (0.6 Mrad) to reduce *Salmonella* spp. (Typhimurium, Senftenberg, Oranienburg, Anatum, Good, and Minnesota) levels by 5 logs. The study also reported that the postirradiation storage did not lead to the recovery of the irradiated *Salmonella*.

In a study by Rana Raj (2006), semimoist pet treats with 10%, 15%, and 25% moisture were treated with gamma irradiation at 2.0, 3.0, 4.0, 6.0, and 8.0 kGy and incubated at room temperature until 180 days. Samples were analyzed at 7, 15, 30, 45, 60, 75, 90, 120, 150, and 180 days. Nondetectable aerobic counts were reported in treats with 10% moisture treated with gamma irradiation doses of 6 and 8 kGy. When it comes to *Salmonella*, even the control treats were negative. Therefore, the irradiation was not indicative of *Salmonella* reduction in this study. Treatment with 4, 6, and 8 Gy of gamma radiation led to nondetectable coliform counts for treats with 10% and 15% moisture; however, in the past 30 days of storage, an increase in coliform counts was detected. In a separate study by Sethukali et al. (2023), commercial semimoist pet foods were exposed to 2.5, 5.0, and 10.0 kGy of electron beam and X‐ray after inoculation with *E. coli* O157:H7 and *S*. Typhimurium. The microbiological evaluation conducted every 20 days showed that the pathogen reduction was better at a higher dosage (10 kGy). However, it also accelerated lipid oxidation and protein degradation compared with the lower dosage (5 kGy) of electron beams and X‐rays.

In a recent study by Kakatkar et al. (2024), a series of experiments were conducted wherein pet food kibble and powders composed of wheat, rice, and the fish by‐product from *Pangasium bocourti* were subjected to gamma irradiation at a dosage of 2.5 and 5 kGy. The investigation revealed a significant extension in shelf life, with 2.5 kGy treated samples exhibiting prolonged viability for 65 days compared with the control (28–35 days). When a higher dosage of 5 kGy of gamma radiation was administered, the total bacterial load remained below the detection limit (< 10 CFU/g) throughout the entirety of the 65‐day observation period. Conversely, in the untreated control samples, whereas *Salmonella* was notably absent, a measurable presence of coliform bacteria (≤ 20 CFU/g) and *Staphylococcus aureus* (ranging between 2.13 and 2.52 CFU/g) was detected. However, upon exposure to 2.5 kGy and 5 kGy of gamma irradiation, the pet food samples exhibited an absence of foodborne pathogens, including *Salmonella*. This underscores the efficacy of gamma irradiation as a means of eliminating potential *Salmonella* contamination in pet food products, thereby enhancing their safety and extending their shelf life.

### 8.2 Biological interventions

#### 8.2.1 Bacteriophage

Due to their specificity, environment‐friendly, and natural abundance, bacteriophages are becoming popular against pathogens in various human foods such as raw meat (Sharma et al., 2015), fresh produce (Lopez‐Cuevas et al., 2021), dairy (Phongtang et al., 2019), seafood (Xu et al., 2018), etc. However, limited research is available on the use of bacteriophages in pet food. The use of bacteriophages in dry foods in general and dry pet food specifically is even more limited, which could be due to the limited growth of bacteria in dry food, making it difficult to locate the phages, and secondly due to the restricted mobilization phage in dry foods.

Heyse et al. (2015) explored the effectiveness of bacteriophages in mitigating *Salmonella* contamination in dried pet food. Pet food samples were inoculated with a cocktail mixture of *S*. Enteritidis, Montevideo, Senftenberg, and Typhimurium at *ca*. 6 logs, followed by thorough mixing to ensure uniform distribution of *Salmonella*. A surface spray of phage preparation to achieve final concentrations of 5, 6, and 7 log PFU/g followed by incubation at room temperature for 1 h led to the *Salmonella* reduction of 0.8, 1.4, and 2.0 log MPN/g, respectively. Similarly, Soffer et al. (2016) evaluated the efficacy of a cocktail bacteriophage consisting of 6 lytic monophages against a *Salmonella* cocktail of Enteritidis, Heidelberg, and Typhi in raw pet foods. Locally purchased raw pet food ingredients such as chicken, turkey, tuna, cantaloupe, and lettuce inoculated with *Salmonella* (*ca*. 1500 CFU/g on chickens; *ca*. 1,250 CFU/g on turkey trim, *ca*. 2000 CFU/g on tuna/cantaloupe; and *ca*. 500 CFU/g on lettuce) followed by a 60‐min attachment time and bacteriophage application. The result showed up to 88%, 68%, 80, 92%, and 89% reduction in chicken, turkey, lettuce, tuna, and cantaloupe with 9×10^6^ PFU/g of bacteriophage. In the case of turkey trim, ∼2×10^7^ PFU/g of bacteriophage was able to cause an 86% reduction in *Salmonella*. The authors also evaluated the effect of bacteriophage‐treated dry pet food kibbles on pets by feeding it to cats and dogs and reported no deleterious side effects in pets. Bacteriophages have been commercially used in fresh pet food, for example, by a company Furchild Pet Nutrition, with a claim to have gained success.

### 8.3 Chemical interventions

Physical interventions, which are point‐in‐time mitigation strategies, may lack carry‐over effects to prevent postprocessing contamination. Meanwhile, the application of chemical additives and antimicrobials usually has the potential to act against pathogens for longer durations (Huss et al., 2017). Therefore, different organic acids, acidulants, medium‐chain fatty acids, and plant‐derived antimicrobial additives are often applied in pet foods as pathogen mitigation interventions.

#### 8.3.1 Liquid smoke

Liquid smoke is a naturally derived flavor component and preservative used in human and pet foods, with known antimicrobial properties (Deliephan et al., 2023a; Lingbeck et al., 2014). Liquid smoke is recognized as a Generally Recognized as Safe (GRAS) additive for human consumption by the U.S. FDA. In the food industry, liquid smoke fractions are used as flavoring agents, browning colorants, antioxidants, texture enhancers, and antimicrobial agents (Deliephan et al., 2023a).

To our knowledge, there is no published study evaluating liquid smoke as an antimicrobial against *Salmonella* in pet food. However, liquid smoke has been used as a flavoring ingredient in a wide range of pet food treats manufactured by major pet food companies, including Blue Buffalo, Purina, Smokehouse Pet Products, and Nutrish. Liquid smoke and its fractions containing phenols, carbonyls, and organic compounds have been found to be effective against pathogenic bacteria like *L. monocytogenes* and *Staphylococcus aureus* in meat and fish products (Lingbeck et al., 2014; Sunen et al., 2003). Though liquid smoke has not been commercially studied in mitigating *Salmonella* in pet food, it has shown antimicrobial activity against fungi in pet food and against *Salmonella* in other food matrices. Therefore, in addition to their use as flavoring agents, the potential use of liquid smoke as an antimicrobial agent in pet foods like dry kibble, semimoist treats, and RMBD needs to be determined.

A study by Deliephan et al. (2023a) evaluated the antifungal effects of liquid smoke fractions against *Aspergillus flavus* in semimoist pet food. Researchers have evaluated liquid smoke fractions in broth assays against *Salmonella* and have proved their inhibitory activity. Kim et al. (2012) reported the minimum inhibitory concentration (MIC) of liquid smoke from rice hull smoke condensate to be 0.822% against *S*. Typhimurium. Another study by Van Loo et al. (2012) evaluated four commercial smoke extracts for which the MICs ranged from 0.5%‐12% against *S*. Typhimurium. Although there is very limited published literature on the sensory palatability of liquid smoke by pets, it can be ascertained that liquid smoke has good application potential in pet foods due to the commercial availability of various kinds of smoke‐flavored pet treats by major pet food companies.

#### 8.3.2 Organic acids and acidulants

There are several organic acids and acidulants commonly used as processing aids or as ingredients in human and animal foods, including lactic acid, citric acid, propionic acid, phosphoric acid, acetic acid (and their salts), and sodium bisulfate (SBS). Most of the organic acids and acidulants are considered GRAS) additives by the U.S. FDA and hence do not have a daily maximum acceptable intake for humans or animals, which increases their applicability in foods. However, their dosage is limited by their negative impact on organoleptic and color attributes of food and meat products in many cases (Kiprotich & Aldrich, 2022).

Nontraditional chicken products, such as hearts and livers, are commonly used in the manufacturing of pet foods and are becoming increasingly popular in RMBD (Cancio et al., 2023). Several studies have looked at *Salmonella* decontamination strategies for these products. Cancio et al. (2023) evaluate the use of peroxyacetic acid (PAA), buffered vinegar, and cultured dextrose fermentate to reduce *Salmonella* on artificially inoculated raw chicken livers intended to be used in pet foods. After immersion, there was a significant *Salmonella* reduction with all treatments, including the water control. More recently, Nakimera et al. (2024) evaluated the efficacy of a blend of citric acid and hydrochloric acid (CP), PAA, and sulfuric acid against *Salmonella* and mesophilic aerobic plate counts (APC) on chicken hearts and livers commonly used in pet food. All antimicrobials reduced *Salmonella* counts by more than one log, in contrast to the water control. The results of these studies demonstrate that *Salmonella* can be mitigated in raw poultry products intended for pet food production using processing aids that are already common in the meat industry.

In the animal feed industry, chemical additives are often derived from blends of organic acids, such as 3‐Hydroxy‐3‐methylbutyrate (HMB), an organic acid available commercially in both free acid (HMBFA) and calcium salt (CaHMB) forms for use in animal feed. HMB functions as a metabolite of the essential amino acid leucine for animals and is recognized as GRAS by the U.S. FDA. Additionally, due to its organic acid properties, it also imparts antimicrobial effects when used in animal food and feed. A study by Huss et al. (2017) evaluated HMBFA or CaHMB as a coating on pet food kibble against *Salmonella*. 1.5% HMBFA reduced *Salmonella* counts by ∼ 4.9 logs in 1 day, whereas 1.5% CaHMB decreased *Salmonella* by *ca*.7.1 in 7 days. All HMBFA and CaHMB treatments reduced *Salmonella* counts to undetectable levels in 14 days. Deliephan et al. (2023b) evaluated two commercial organic acid mixtures containing hydroxy‐4‐(methylthio) butanoic acid (HMTBa) at 2% and 1%, respectively, as a coating on kibble inoculated with *Salmonella* or *E. coli* O157:H7 (STEC). *Salmonella* counts were reduced by *ca*. 3 logs after 12 h and up to 4.6 logs after 24 h. STEC counts were also reduced by *ca*. 2 and 3 logs after 12 h and 24 h, respectively. Similarly, O'Bryan et al. (2015) evaluated a proprietary mixture of an organic acid blend consisting of 5%–25% nonanoic acid, 1%–25% butyric acid, and 1%–50% trans‐2‐hexenal on meat and bone meal (commonly used as a pet food ingredient) at 0, 1, 1.5, or 2 mL/kg of feed. Microbial analysis over time resulted in about 1 log reduction of *Salmonella* by 24 h and *ca*. 2 log reduction in 14 days.

SBS is another GRAS acidulant approved for use as an additive in human and animal foods by the U.S. FDA. Due to its hygroscopicity and desiccant effect, SBS is found to be effective in killing pathogens such as *Salmonella* and *Campylobacter* (Dhakal & Aldrich, 2021; Line, 2002). SBS is commonly used in animal diets for the acidification of feline urine and for the preservation of soft‐moist treats and liquid digests. Dhakal et al. (2019b) evaluated SBS, lactic acid, phosphoric acid, and combinations of butyric and propionic acids in rendered chicken fat (used to coat dry pet food kibbles) inoculated with *Salmonella*. SBS or lactic acid at 0.5% individually or a combination of SBS with propionic and butyric acid reduced *Salmonella* loads by >5.5 logs within 15 h in the chicken fat without negatively altering the shelf life of rendered fat (Dhakal et al., 2019). Dhakal and Aldrich (2023) evaluated the acidulants SBS, phosphoric acid, and lactic acid, individually and in combination with organic acids butyric and propionic acid in different fat types, namely, chicken fat, canola oil, Menhaden fish oil, lard, and tallow that are intended to coat dry pet food kibbles. The treated fats were inoculated with approximately 8 logs of *Salmonella*. SBS at 0.5%, phosphoric acid at 0.5%, and lactic acid at 0.25% individually and in combination with butyric acid at 0.075% and propionic acid at 0.05% reduced *Salmonella* loads below detectable limits within 2 h across all fats. The highest antibacterial efficacy was observed in Menhaden fish oil, with the *Salmonella* loads reduced to below detectable limits in less than 1 h.

#### 8.3.3 Fatty acids

Medium and long‐chain fatty acids are considered effective antimicrobial feed additives in animal feed. Research by Cochrane et al. (2016) demonstrated the antimicrobial effects of medium‐chain fatty acids against *Salmonella* in rendered protein meals used in the animal feed industry. In pet food research, medium‐chain fatty acids, namely, caproic (C6), caprylic (C8), and capric (C10) acids, were evaluated by Dhakal and Aldrich (2020) as coating on dry kibbles inoculated with *Salmonella*. C6, C8, and C10 at 0.5%–1% reduced *Salmonella* levels by >4.5 logs after 5 h of treatment. A combination of C6 + C8 (0.25%–0.5%) reduced *Salmonella* levels to below the detection limit in 4 h, whereas C6 + C10 (0.25%–1%) and C8 + C10 (0.25%–1%) did the same in 2–4 h and 1–5 h, respectively, displaying potential synergism. Although the MCFA was effective against *Salmonella* in pet food, MCFA‐coated dry dog kibbles did not enhance the palatability of the diets, and dogs preferred control diets over the MCFA‐coated diets. On the other hand, fish oils are rich sources of long‐chain omega‐3 fatty acids and are used as human dietary supplements and as pet food ingredients. Menhaden fish oil is a long‐chain omega‐3 fatty acid and a popular commercial pet food ingredient rich in polyunsaturated fatty acids (PUFA). PUFAs from fish sources have shown antibacterial activity against several pathogenic microorganisms, including *E. coli*, S. *aureus*, and *Salmonella* (Chitra Som & Radhakrishnan, 2011). Dhakal and Aldrich (2022) evaluated Menhaden fish oil in vitro and as a coating on dry pet food kibble against *Salmonella* at different storage temperatures of 25°C, 37°C, and 45°C. *Salmonella* levels in the fish oil were below detection limits by 2 h at all temperatures. On the kibble, the fish oil had higher antimicrobial activity after 12 h at 25°C and after 2 h at 45°C, thus increasing with temperature. Overall, higher antimicrobial activity of the fish oil was observed at 37°C and 45°C throughout the experiment, indicating that higher holding temperatures could enhance the antimicrobial efficacy of Menhaden fish oil.

#### 8.3.4 Plant‐derived antimicrobials

Plant‐derived antimicrobials (PDA), such as trans‐cinnamaldehyde, carvacrol, thymol, eugenol, and caprylic acid applied as vegetable oil or chitosan‐based antimicrobial spray on pet food kibble for reducing *Salmonella* were investigated by Chen et al. (2019). All PDAs at 1% and 2% applied in vegetable oil or chitosan reduced *Salmonella* by at least 2 log CFU/g in 3 days compared with the control. Trans‐cinnamaldehyde at 2% was the most effective, with a 3–3.5 log CFU/g reduction of *Salmonella* during storage. Kiprotich et al. (2021) treated *Salmonella*‐inoculated raw chicken breast meat with thyme oil at 0.5% (v/v) added into lemon juice and supplemented with *Yucca schidigera* extract, a natural emulsifier, at 23°C for 8 h. The 0.5% thyme oil treatment resulted in a 3.48 log reduction of *Salmonella* in 8 h. Boskovic et al. (2017) combined thyme oil treatment at 0.3% along with vacuum packaging on minced pork meat, a common ingredient of raw pet food stored under refrigeration at 3 ± 1°C for 15 days. About 1.7 log reduction of *Salmonella* counts was observed by 15 days. Similarly, Thanissery and Smith (2014) combined thyme oil and orange essential oil at 0.5% (v/v) each and achieved a 2.6 log reduction of *Salmonella* and a 3.6 log reduction of *Campylobacter coli* in chicken breast meat, another commonly used ingredient in raw pet food diet.

A broader commercial application of PDA in pet foods could be its cost‐effectiveness. Additionally, as mentioned earlier, another limitation of some organic acids, acidulants, and fatty acids in pet foods is their impact on sensory attributes like taste, aroma, and flavor due to their low pH, high acid, and strong smell. Similarly, the strong smell and taste of PDA like essential oils are limitations in their application. In these cases, a “slow‐release mechanism” of these ingredients through encapsulation technologies could be an alternative. For instance, encapsulating organic acids with soluble and edible vegetable oil films allows for a slow release of the acid into the food product at a controlled rate, thereby minimizing organoleptic impact in terms of flavor and taste (Kiprotich & Aldrich, 2022).

#### 8.3.5 Ozone treatment

Ozone treatment employs a chemical method where contaminated food products are exposed to ozone in either an aqueous or gaseous phase. When ozone molecules create oxidative reactive species, they rupture the cell wall and damage the cell wall proteins, enzymes, and nucleic acids (Brodowska et al., 2018; Cano et al., 2019). The excess ozone rapidly decomposes to oxygen, thus leaving no toxic residues in food. The treatment with ozone requires no thermal energy, making it suitable for heat‐sensitive products, and the exclusion of heat generation saves the need for input energy (Cano et al., 2019; Kaavya et al., 2021).

Ozone has been used to reduce *S*. Typhimurium, *E. coli*, and *Listeria innocua* contamination in fruits and vegetables such as cilantro, strawberries, romaine lettuce, and tomatoes (Alexopoulos et al., 2013; Chandran et al., 2023; Chen et al., 2019; Gibson et al., 2019). In a study by Chandran et al. (2023), the effectiveness of a spray and batch wash ozone system (5 ppm) against *Salmonella* and *L. monocytogenes* on surfaces of carrots, sweet potatoes, and butter squash commonly used in RMBD was evaluated. The batch wash system resulted in up to 1.56 CFU/mL mean microbial reduction; however, this was not significantly different from the control. Meanwhile, with the spray wash system, freeze‐tempered produce showed a higher bacterial reduction with 5 ppm ozone than the control but was not different from room temperature produce. Ozone gas is also used to decontaminate *Aspergillus flavus* spores in extruded pet foods. Silva et al. (2018) reported a reduction of up to 98.3% of inoculated spores after 120 min exposure to 40 or 60 µmol/mol ozone and 84% reduction after 30 min at 40 µmol/mol ozone. *Salmonella* decontamination of raw chicken parts using ozonated water has only seen minimal reductions (Cano et al., 2023). However, ozone‐based treatment of pet food products has not been extensively studied, leaving a research opportunity.

## 9 REGULATORY MEASURES IN PET FOODS

**FIGURE 3:**
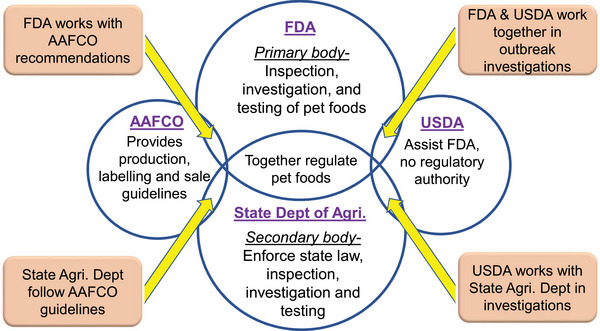
Collaborative roles of different agencies in maintaining safe and quality pet foods (Thixton, 2017).

In the United States, pet foods are subject to extensive regulation, making it one of the highly regulated food products to which compliance with both federal and state regulations is mandatory (Pet Food Institute, 2023). As shown in Figure 3, there are four major bodies involved in pet food safety and quality. Firstly, the Association of American Feed Control Officials (AAFCO) provides guidelines for the production, labeling, and sale of pet food and related products and is not involved in testing, regulating, and certifying the pet food to ensure it meets standards (AAFCO, 2024b). Secondly, the U.S. FDA regulates and monitors the safety of pet food products, including their ingredients, labeling, and production processes (FDA, 2024c). FDA establishes and enforces standards for pet food under the Federal Food, Drug, and Cosmetic Act. The FDA conducts inspections, investigates complaints, and issues recalls if necessary to address safety concerns. The Center for Veterinary Medicine (CVM) is a branch of the FDA specifically focused on veterinary medicine, including the regulation of animal food and drugs. It works to ensure the safety and efficacy of products intended for animals, including pet food. The CVM conducts research, establishes regulations, and collaborates with other agencies and stakeholders to address emerging issues in the pet food industry. Each state department of agriculture has its own requirements, regulations, and jurisdiction regarding the label and safety of pet foods.

With the passage of FSMA in 2011, new mandatory product safety standards are required for almost all U.S. pet food producers. The Preventive Controls for Animal Foods rule (21 CFR 507) requires pet food makers to implement current GMP (cGMPs), identify and evaluate biological, chemical, and physical hazards, develop and implement proper food safety plans detailing the steps they are taking to ensure product safety. Overall, the FSMA provisions to develop, implement, and maintain sanitary standards and robust verification activities, including environmental monitoring when needed, are expected to help reduce the burden of pet food contamination leading to product recalls and potential outbreaks.

It is deemed unacceptable for pet foods to contain *Salmonella*, as such products would be classified as adulterated under CPG Sec. 690.800 (FDA, 2013d). The same regulation is also observed in the European Union based on E.U. Commission Regulation No. 142/2011, Annex XIII. However, in Canada, the requirement about the presence of *Salmonella* is not implicit. The regulation of pet food by the state departments of agriculture is influenced by state laws, always in collaboration with federal laws. Although pet food is monitored by the FDA, it does not strictly enforce all the laws that are required for pet food and animal feed. Most of the states in the United States require pet food manufacturers to register every year for the foods and treats they sell within the state (APPA, 2024). Some states infrequently inspect adherence to labeling requirements and randomly test pet foods for microbial safety compliances, whereas some states only investigate consumer complaints along with the FDA. Although pet foods are monitored by the FDA, the frequency of routine inspections is done at least once every three years for domestic high‐risk facilities and at least once every five years for non–high‐risk facilities. They also conduct targeted inspections. However, this is conducted in relation to an outbreak, factors posing contamination risks, food consumption patterns, regional influences, trends, and compliance history of the manufacturer (FDA, 2024b).

Meat and by‐product meals known to be unfit for human consumption from USDA‐inspected meat processing facilities have been used by pet food and animal feed manufacturing plants in FDA‐inspected operations. In one notable example, the FDA initiated a recall of canned dog food from Evanger's Dog & Cat Food Company in 2017 due to phenobarbitol being traced back and found to have originated from a ‘USDA‐approved’ meat supplier. However, further investigation revealed that the lot was USDA labeled as “Inedible hand deboned beef‐ for pet food use only; Not fit for human consumption.” This indicates that pet food safety regulations are not as stringently enforced as human food regulations.

## 10 CONCLUSION

**FIGURE 4:**
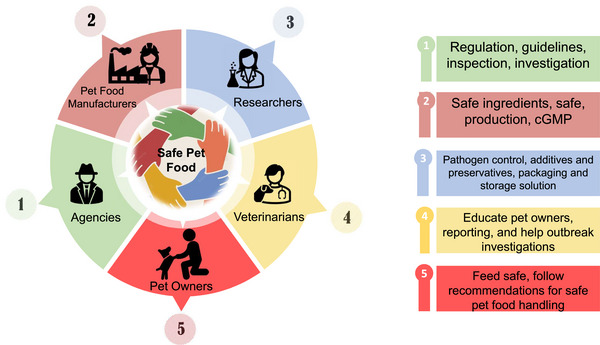
Collective efforts in maintaining safe and quality pet foods.

As the pet population and pet food market are increasing, they are boosted by the increasing humanization and premiumization of pets. Pet owners’ preferences and perceptions play a big role in the choice and type of feed a pet receives. However, as the owner's choice drives the new and emerging products and feed types in the pet food industry, it is crucial not to compromise the health of pet owners for the sake of pet food advancements. Given the recent rise in *Salmonella*‐linked recalls in pet food, particularly RMBD, and the concurrent increase in human cases, especially among children and the elderly, a multifaceted approach is necessary. This approach should involve the pet food industry, consumer education, researchers, veterinarians, and policymakers to safeguard the health of both pets and their owners (Figure 4).

Despite the lack of standardized pathogen elimination steps in pet food production, several measures can mitigate *Salmonella* contamination. Prevention of cross‐contamination and postprocessing contamination in dry pet food can help to safeguard dry pet foods. The use of approved interventions such as processing aids, GRAS chemicals, and biological methods could be an alternative to mitigate *Salmonella* contamination in raw pet foods. Additionally, implementing current GMP (cGMPs) and proper Hazard Analysis Critical Control Point plans at manufacturing facilities can significantly enhance the safety of pet food products. Educating pet owners about the potential risks associated with product handling, cleaning, hand washing, and sanitation, as well as the risk of carrier pets and *Salmonella*, can help reduce the incidences of human *Salmonella* outbreaks linked to pet foods. Proper storage and handling of pet foods, maintaining appropriate temperature and relative humidity, and ensuring the quality and cleanliness of raw ingredients are essential practices for keeping pet food safe.

## AUTHOR CONTRIBUTIONS

**Janak Dhakal**: Conceptualization; writing–original draft; methodology; data curation; investigation; funding acquisition; project administration. **Leslie Cancio**: Investigation; writing–review and editing; validation; resources; data curation. **Aiswariya Deliephan**: Investigation; writing–review and editing; validation; resources. **Byron Chaves**: Review and editing; validation. **Stephan Tubene**: Review and editing; validation.

## FUNDING

This work is supported by the Capacity Building Grant [project award no. 2024‐38821‐42105], from the U.S. Department of Agriculture's National Institute of Food and Agriculture (USDA‐NIFA).

## CONFLICTS OF INTEREST STATEMENT

The authors have no conflict of interest to declare.
